# Genomic Insights Into Species Divergence and Adaptive Evolution in the Grass Genus *Orinus* on the Qinghai‐Tibet Plateau

**DOI:** 10.1002/ece3.73641

**Published:** 2026-05-10

**Authors:** Ai Liu, Xue Yang, Juan Lu, Kunjing Qu, Jinyuan Chen, Xu Su, Guangpeng Ren, Hongyin Hu

**Affiliations:** ^1^ College of Geology and Jewelry Lanzhou Resources & Environment Voc‐Tech University Lanzhou China; ^2^ Yellow River Basin Ecotope Integration of Industry and Education Research Institute Lanzhou Resources & Environment Voc‐Tech University Lanzhou China; ^3^ State Key Laboratory of Herbage Improvement and Grassland Agro‐Ecosystems, College of Ecology Lanzhou University Lanzhou China; ^4^ Key Laboratory of Biodiversity Formation Mechanism and Comprehensive Utilization of the Qinghai‐Tibet Plateau in Qinghai Province Qinghai Normal University Xining China; ^5^ Academy of Plateau Science and Sustainability Qinghai Normal University Xining China

**Keywords:** adaptive evolution, genomic islands, *Orinus*, Quaternary climatic oscillations, speciation with gene flow

## Abstract

The grass genus *Orinus*, endemic to the Qinghai‐Tibet Plateau (QTP), has long been taxonomically controversial. Recent integrative studies combining morphological and DNA barcoding proposed revising the genus from six to three species; however, this hypothesis lacks validation from genome‐wide data, and the evolutionary history of the putative species remains poorly resolved. Here, we conducted whole‐genome resequencing of 126 individuals from 40 populations representing three putative species of *Orinus*—*Orinus thoroldii*, *Orinus kokonorica*, and *Orinus intermedius*—and performed population genomic and demographic analyzes. Genome‐wide evidence robustly supports the three‐species delimitation and reveals pronounced genetic differentiation among lineages. We further detected both historical and recent hybridization between *O. kokonorica* and *O. intermedius* , indicating incomplete reproductive isolation. Demographic inference suggests that divergence among lineages occurred during Quaternary climatic oscillations, when repeated climate fluctuations, together with the complex topography of the QTP, likely promoted lineage divergence while allowing persistent and asymmetric gene flow, particularly during early stages of divergence. Across species pairs, we consistently identified genomic islands of divergence characterized by elevated absolute nucleotide divergence (*D*_xy_). Genes located within these regions show signatures of positive selection and are enriched in functions related to environmental adaptation, suggesting a role of natural selection in maintaining species boundaries. Together, our results provide robust genomic support for the revised taxonomy of *Orinus* and offer new insights into how Quaternary environmental dynamics have shaped speciation and genomic divergence in a montane grass lineage.

## Graphical abstract

Using population genomic data, we investigated the evolutionary history and genomic divergence of closely related terrestrial plant lineages to test whether speciation proceeded in the presence of gene flow. Demographic modeling and genome‐wide scans revealed divergence with ongoing gene flow, heterogeneous genomic differentiation, and signatures of selection associated with environmental adaptation. Our results highlight the joint roles of historical climatic fluctuations, gene flow, and natural selection in shaping plant diversification.

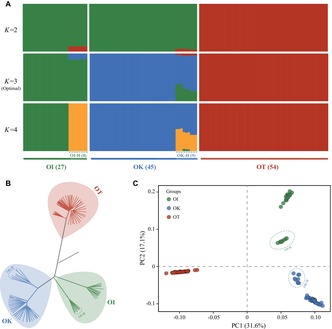

## 1 Introduction

Speciation, a fundamental driver of biodiversity, remains a central topic in evolutionary biology. Understanding the modes, processes, and genetic mechanisms that generate new species is critical for explaining patterns of biodiversity (Ellegren et al. 2012; Lawniczak et al. 2010; Seehausen et al. 2014). Darwin (1859) first emphasized the role of natural selection in promoting population divergence, and classical evolutionary theory later posited that, in the absence of gene flow, genetic drift and divergent selection can drive the fixation of alternative alleles in geographically isolated populations, ultimately resulting in genetic incompatibilities and allopatric speciation (Bateson 1909; Mayr 1942). However, mounting evidence over the past two decades indicates that speciation is far more dynamic than this strictly allopatric model predicts. Numerous empirical studies now show that divergence can arise and persist in the presence of gene flow, and that closely related species often harbor signatures of both ancient introgression and recurrent hybridization (Hu et al. 2022; Li et al. 2021; Ma et al. 2018; Martin et al. 2013). Such complexity is expected in regions where climatic and geographic processes cause repeated shifts in species distributions. During the Quaternary, cyclical glacial–interglacial oscillations repeatedly drove range contractions and expansions, creating opportunities for both isolation and secondary contact (Hewitt 2000, 2004). When reproductive barriers are incomplete, secondary contact between nascent lineages frequently leads to hybridization, hybrid backcrossing, and introgression of differentiated alleles across species boundaries, potentially obscuring biogeographic and genomic signals of divergence. Consequently, reconstructing divergence times and the spatiotemporal dynamics of interspecific gene flow is essential for clarifying how historical environmental change interacts with demographic processes and gene flow to shape the speciation process (Network 2012).

Localized genomic regions exhibiting exceptionally high divergence, commonly referred to as “genomic islands”, have traditionally been interpreted as barriers to gene flow and as candidate regions contributing to the evolution of reproductive isolation between incipient species (Abbott et al. 2013; Wu 2001). In this framework, gene flow homogenizes the remainder of the genome, thereby constraining divergence outside these islands (Feder et al. 2012; Nosil et al. 2009). However, an increasing number of studies indicate that genomic islands may arise through processes unrelated to adaptation or speciation (Guerrero and Hahn 2017; Wolf and Ellegren 2017). Heterogeneous genomic divergence between closely related species can instead be shaped by multiple mechanisms, including the differential sorting of ancestral polymorphisms, divergence hitchhiking, and genomic features such as variation in recombination rate and gene density (Ke et al. 2022; Wang et al. 2020). Moreover, lineage‐specific selective sweeps driven by environmental adaptation can produce peaks of elevated divergence both within and outside genomic islands (Hu et al. 2021, 2022; Ma et al. 2018), highlighting that species boundaries emerge from the combined actions of diverse evolutionary processes (Hoskin et al. 2005; Karrenberg et al. 2019; Ren et al. 2018). Despite these advances, the genetic architecture and relative contributions of these processes remain poorly resolved in many taxa.

In this study, we investigate the speciation dynamics of *Orinus*, an endemic grass genus restricted to the Qinghai‐Tibet Plateau (QTP). As the world's highest (mean elevation > 4000 m) and largest plateau, the QTP provides an exceptional setting for examining how geological and climatic processes drive biodiversity. Its history of rapid Miocene–Pliocene uplift and pronounced Quaternary climatic oscillations has repeatedly reshaped species distributions and diversification across the region (Liu et al. 2014; Ren et al. 2015, 2017; Wu et al. 2022). For closely related taxa, such climatic cycles can produce alternating phases of isolation and secondary contact, facilitating extensive hybridization and potentially leading to taxonomic misclassification when based solely on morphological characters. Historically, *Orinus* was classified into six species, three widespread and three narrowly distributed, yet species delimitation often relied on limited morphology‐based specimen comparisons (Su et al. 2015). When such specimens originate from hybrid zones where reproductive isolation is incomplete, putative species may instead represent hybrids of sister taxa, raising doubts about the accuracy of traditional classifications.

To address these uncertainties, Su et al. (2015) applied integrative approaches combining DNA barcoding, morphology, and ecological niche models to revise the taxonomy of *Orinus*, reducing six species to three: *Orinus thoroldii*, *Orinus kokonorica*, and *Orinus intermedius* (Su et al. 2015). The intermediate morphology and broad sympatry of *O. intermedius* further suggest a possible hybrid origin involving the other two species; however, this hypothesis remains unresolved because conventional molecular markers lack sufficient phylogenetic resolution. Consequently, the evolutionary relationships among these three taxa, the extent and timing of gene flow, and the genomic landscape of divergence remain unclear. Building on this taxonomic revision, we generated whole‐genome resequencing data to test whether the revised three species represent cohesive genomic lineages and to resolve their divergence and introgression history. Specifically, we aimed to determine (i) when the three species diverged, whether hybridization occurred, and whether gene flow accompanied divergence, and (ii) how genomic differentiation is structured across the genome and whether positively selected genes within genomic islands are linked to environmental adaptation.

## 2 Materials and Methods

### 2.1 Sample Collection and Whole Genome Resequencing

Following recent taxonomic revisions within *Orinus* (Su et al. 2015), we treated *O. thoroldii* (OT), *O. kokonorica* (OK), and *O. intermedius* (OI) as three distinct species. Extensive field surveys were conducted across the QTP and adjacent regions, covering 40 natural populations. For each population, 8–15 individuals were sampled, maintaining a minimum distance of 20 m between sampled plants to avoid collecting close relatives or clonal ramets. Leaf tissues were dried in silica gel and stored for DNA extraction. For whole‐genome resequencing, three individuals were selected from each OT and OK population, and four from each OI population (except OI03, with three individuals). In total, 126 individuals were chosen for sequencing (Table S1), and *Cleistogenes songorica* was included as an outgroup. Whole‐genome resequencing was performed on the Illumina HiSeq X10 platform using 150 bp paired‐end libraries.

### 2.2 Single‐Nucleotide Polymorphism (SNP) Calling and Filtering

High‐quality paired‐end reads were obtained using FASTP v0.20.0 (Chen et al. 2018), which removed adapter sequences and low‐quality bases. Clean reads were aligned to the *O. kokonorica* reference genome (Qu et al. 2023) using BWA‐MEM v0.7.17‐r1188 with default parameters (Li and Durbin 2009). PCR/optical duplicates were removed using SAMtools v1.18 (Li et al. 2009). Variant calling followed the GATK Best Practices pipeline v4.6.0.0 (DePristo et al. 2011). HaplotypeCaller was used to produce per‐sample GVCF files, which were subsequently joint‐genotyped using GenotypeGVCFs. Raw SNPs were extracted using the SelectVariants module in GATK. To obtain a high‐confidence SNP dataset, site‐level hard filtering was applied using the following GATK thresholds: QD < 2.0, FS > 60.0, MQ < 40.0, MQRankSum < −12.5, and ReadPosRankSum < −8.0. Additional genotype‐ and sample‐level filters were then applied, retaining only SNPs that satisfied all of the following criteria: (i) located ≥ 5 bp from any predicted indel; (ii) genotype quality (GQ) ≥ 30; (iii) biallelic; (iv) genotype depth within the 2.5% to 97.5% of the genome‐wide depth distribution; (v) minor allele frequency (MAF) ≥ 0.05; (vi) genotyped in ≥ 80% of individuals assessed using VCFtools v0.1.16 (Danecek et al. 2011).

### 2.3 Phylogenetic Reconstruction, Ancestry Inference, and Historical Gene Flow

To characterize genetic structure, we combined PCA, ADMIXTURE, neighbor‐joining phylogeny, IBD analysis, and TreeMix inference based on the SNP dataset. PCA was performed using SMARTPCA v18140 in EIGENSOFT (Patterson et al. 2006). ADMIXTURE v1.3.0 analyzes were performed for *K* = 2–6 using the cross‐validation procedure (‐‐cv ‐B 100 ‐j 10) (Alexander et al. 2009). The number of ancestral clusters was evaluated from the CV‐error profile, and the optimal K was selected as the smallest value after which further increases in K produced only marginal decreases in CV error. Pairwise genetic distances were computed in PLINK v1.90b6.18 and used to construct a neighbor‐joining tree in PHYLIP v3.697 (Purcell et al. 2007). IBD segments were identified using BEAGLE v5.5 with window = 100,000, overlap = 10,000, ibdtrim = 100, and ibdlod = 10 (Browning and Browning 2007). Historical population splits and migration were inferred using TreeMix v1.13 (Pickrell and Pritchard 2012). SNP allele counts were generated with treemix.pl., and TreeMix was run using a 500 SNP block size (−k 500), allowing the number of migration edges (m, i.e., discrete migration events) to vary from 0 to 5. For each value of m, 100 bootstrap replicates were performed to evaluate the stability of the inferred migration edges and overall tree topology.

### 2.4 Demographic Inference Analysis

We reconstructed the demographic history of the three species using the Pairwise Sequentially Markovian Coalescent (PSMC) v0.6.5‐r67 model (Li and Durbin 2011). Diploid consensus sequences were generated using the *samtools mpileup* command (Danecek et al. 2011), and PSMC was run with 100 bootstrap replicates using the parameters “‐N25 ‐t15 ‐r5 ‐p ‘4+25*2+4+6’” to obtain 95% confidence intervals. Given that all three species are perennial, we assumed a generation time of 2 years when scaling PSMC time estimates. The mutation rate (μ) was estimated with *r8s* v1.81 (Sanderson 2003) based on fossil‐calibrated divergence times.

To complement the PSMC analysis, we inferred demographic parameters under alternative divergence scenarios using a composite‐likelihood, coalescent simulation framework implemented in fastsimcoal2 v2.6.0.3 (Excoffier et al. 2021). Folded joint site frequency spectra (SFS) were estimated separately using realSFS in ANGSD v0.933 (Korneliussen et al. 2014) from genotype likelihoods calculated directly from BAM files. We stratified the procedure into two steps. First, we set three models to determine the most likely species tree topology (Figure S5). Second, based on this topology, we tested 12 scenarios of gene flow among the three species (Figure S6). For each demographic model, we performed 100 independent runs, each consisting of 100,000 coalescent simulations (−n100000 ‐N100000) and 40 cycles of the conditional maximization algorithm (−L 40) to ensure convergence to the global maximum likelihood estimate. Model choice was based on Akaike's Information Criterion (AIC). Confidence intervals for parameter estimates were obtained from 100 parametric bootstrap datasets, each analyzed by 50 independent fastsimcoal2 runs under the best‐fitting model.

### 2.5 Linkage Disequilibrium, Recombination, Genomic Diversity, and Divergence

We quantified patterns of linkage disequilibrium (LD) and recombination rate variation among the three species. Pairwise LD was estimated as the squared correlation coefficient (*r* ^*2*^) between SNPs using PopLDdecay v3.41 (Zhang et al. 2018), and LD decay curves were generated by averaging *r* ^*2*^ values across pairwise SNPs stratified by physical distance. To minimize the influence of unequal sample sizes, each species was randomly subsampled to an equal number of individuals (*n* = 27). Population‐scaled recombination rates (ρ = 4*Ne*r) were estimated from intraspecific polymorphism data using FastEPRR2 v2.0 (Hao et al. 2022) under a sliding‐window framework (window size = 20 kb; step size = 5 kb). Genomic summaries of nucleotide diversity (π), genetic differentiation (*F*_ST_), and absolute sequence divergence (*D*_xy_) were computed using popgenWindows.py (https://github.com/simonhmartin/genomics_general), applying non‐overlapping 20 kb windows for all three statistics.

### 2.6 Genomic Islands of Divergence

We identified genomic regions showing exceptional levels of interspecific differentiation using a combined empirical and permutation‐based framework (Ma et al. 2018). To avoid the confounding effects of recent admixture on divergence estimates, individuals identified as admixed in the admixture analysis were excluded. Genome‐wide *F*_ST_ was calculated for non‐overlapping 50 kb windows. To account for heterogeneity in the number of SNPs per window, we generated a null *F*_ST_ distribution for each window size category by randomizing SNP positions across the genome while preserving the number of segregating sites per window. For each window, an empirical *p*‐value was obtained by comparing its observed *F*_ST_ value against the corresponding null distribution, and *p*‐values were adjusted for multiple testing using the Benjamini‐Hochberg false discovery rate (FDR). Windows were considered outliers if they fell within the top 5% of the empirical *F*_ST_ distribution and had FDR‐adjusted *p* < 0.01. This dual criterion reduces biases associated with fixed divergence thresholds and mitigates stochastic variation caused by windows containing few informative sites. Adjacent outlier windows were subsequently merged to define contiguous genomic islands of divergence.

### 2.7 Genes Under Positive Selection

We identified lineage‐specific signals of positive selection using the Hudson‐Kreitman‐Aguadé (HKA) test (Hudson et al. 1987) and the population branch statistic (PBS) (Yi et al. 2010). For each species, we quantified (i) the number of segregating sites within the focal lineage and (ii) the number of fixed differences between the focal lineage and the other two species, defined as sites at which the compared species are reciprocally fixed for alternative alleles. HKA tests were performed for each gene by comparing its ratio of polymorphism to divergence with the genome‐wide expectation. To control for multiple testing across gene‐wise HKA analyzes, the resulting *p* values were adjusted using the Benjamini‐Hochberg false discovery rate (FDR) procedure. To complement the HKA analysis, we computed pairwise *F*_ST_ values among species and transformed them into lineage‐specific PBS values, which reflect the amount of divergence accumulated along each lineage. Genes with FDR‐adjusted HKA *p* values < 0.01 and PBS values within the top 5% of the empirical distribution were considered candidate positively selected genes (PSGs). Candidate genes were annotated and functionally classified based on Gene Ontology (GO) categories, and enrichment tests were performed using Fisher's exact test with Benjamini‐Hochberg correction (*FDR* < 0.05).

## 3 Results

### 3.1 Genome Resequencing and Population Structure of *Orinus*

**FIGURE 1:**
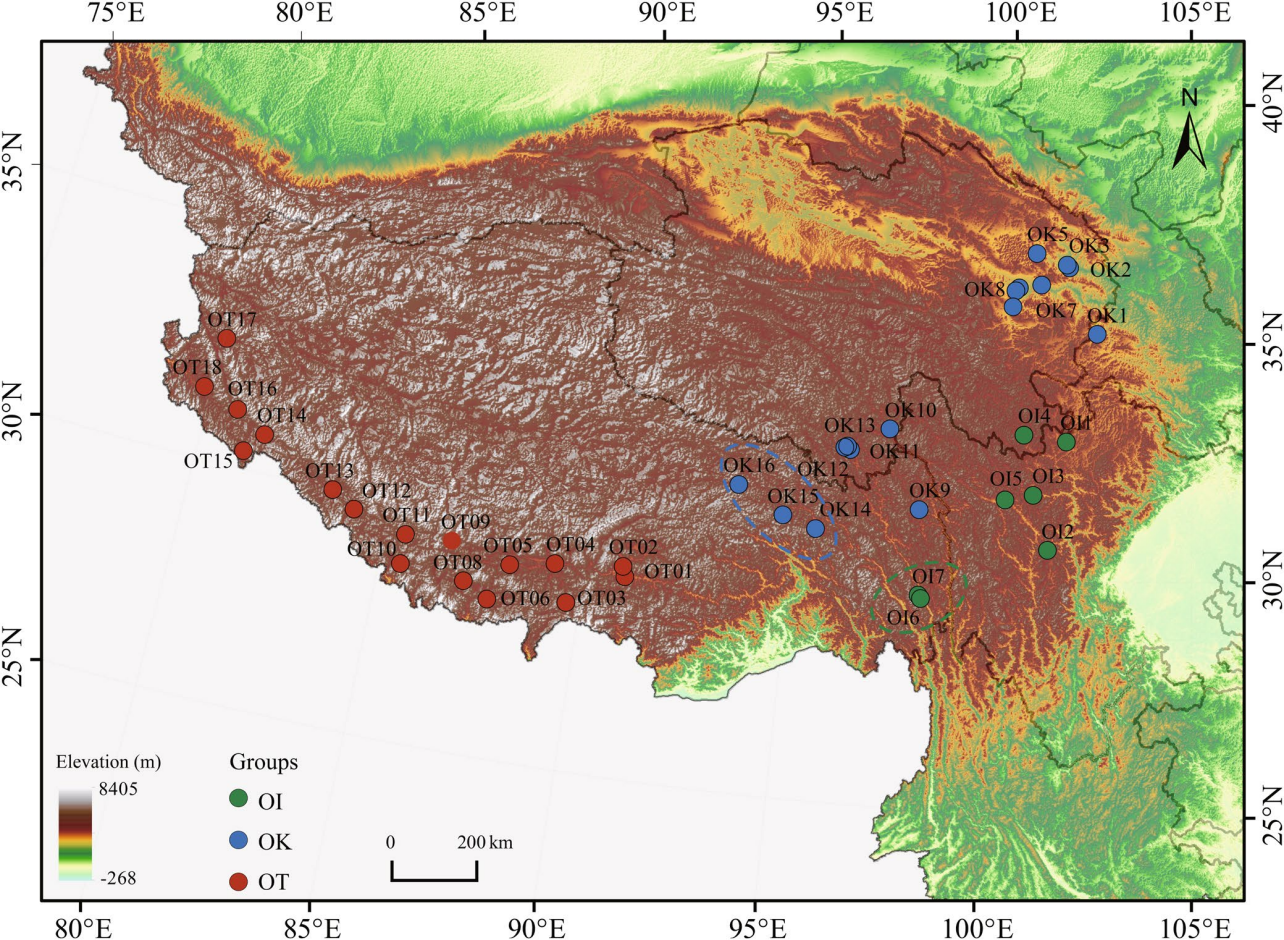
Geographic distribution of sampled *Orinus* populations across the QTP. Each point represents one sampled population, color‐coded by species: *O. intermedius* (OI; green), *O. kokonorica* (OK; blue), and *O. thoroldii* (OT; red). Dashed circles indicate geographically localized population groups containing admixed individuals identified by ADMIXTURE, including OI6‐OI7 and OK14‐OK16. The background shows the topographic elevation gradient of the region. The map illustrates the distinct geographic ranges of the three species, with OT primarily distributed along the Himalayan region, OK in the northeastern QTP, and OI in the northern Hengduan Mountains.

We resequenced 54 *O. thoroldii* (OT), 45 *O. kokonorica* (OK), and 27 *O. intermedius* (OI) individuals sampled from 40 populations spanning their entire geographic ranges on the QTP (Figure 1; sample information detailed in Table S1). After quality control, clean reads were aligned to the *O. kokonorica* reference genome, resulting in an average effective depth of 17.49× and a mean mapping rate of 80.48% per individual (Table S1). SNP calling followed the GATK4 best‐practice workflow. HaplotypeCaller infers genotypes based on genotype likelihoods (GLs), and stringent variant filtering was applied to obtain high‐confidence SNPs. In total, 6,316,026 high‐quality SNPs were retained for downstream population genetic analyzes. Although using the *O. kokonorica* reference genome for all three species may introduce some reference bias, the moderate missingness observed after filtering suggests that any residual bias was limited (Table S2).

**FIGURE 2:**
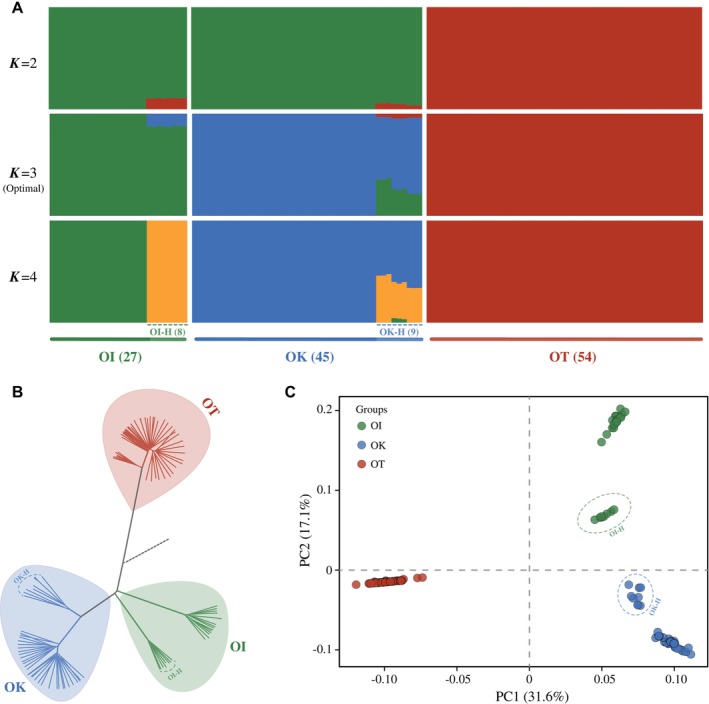
Population genetic structure of three *Orinus* species based on genome‐wide SNPs. (A) Individual ancestry proportions inferred by ADMIXTURE for *K* = 2–4 (*K* = 3 identified as the best model). Each vertical bar represents one individual, and colors indicate the inferred genetic clusters. Individuals are grouped by genetic clusters corresponding to OI, OK and OT (sample sizes in parentheses). The subsets labeled OI‐H and OK‐H represent hybrid individuals. (B) Neighbor‐joining (NJ) tree constructed from pairwise genetic distances calculated in PLINK and reconstructed in PHYLIP. Individuals form three well‐supported genetic clusters corresponding to OK, OI, and OT. *Cleistogenes songorica* was used as an outgroup. (C) Principal component analysis (PCA) illustrates clear separation among the three species along PC1 (31.6%) and PC2 (17.1%). The minor subclusters within OI and OK in the NJ tree and PCA correspond to geographically restricted admixed populations (OI‐H: OI6‐OI7 and OK‐H: OK14‐OK16) and do not represent additional taxonomic units.

We employed ADMIXTURE, phylogenetic reconstruction, and PCA to assess the genetic relationships among individuals, which revealed an evolutionary history different from previous morphological‐based taxonomy (Su et al. 2015). Across analyzes, genome‐wide data consistently supported three primary genetic lineages corresponding to OT, OK, and OI. ADMIXTURE revealed clear structure across *K* values (Figures 2A and S1). At *K* = 2, OT was clearly separated from the other two species, whereas several individuals in OK (populations OK14‐OK16) and OI (populations OI6‐OI7) showed admixture. At the optimal *K* = 3, OK and OI formed two distinct genetic clusters, consistent with their species delimitation and overall geographic separation. The eight admixed OI individuals carried approximately 16% ancestry from OK, whereas the nine admixed OK individuals showed mixed ancestry from multiple components. At *K* = 4, the OI admixed individuals formed a distinct fourth cluster, whereas the OK admixed individuals remained genetically mixed. The CV‐error profile showed that the decrease in CV error became marginal after *K* = 3 (Figure S1).

The NJ tree and PCA recovered the same overall pattern: OT formed a deeply divergent lineage, while OK and OI were clearly separated but each contained a minor internal subgroup (Figure 2B,C). Importantly, these within‐species subgroups correspond to geographically restricted populations (OI6‐OI7 and OK14‐OK16; Figure 1) that also show admixture in ADMIXTURE, indicating phylogeographic structure combined with localized introgression rather than additional taxonomic entities. PC3 (9.9% of the variance) further captured within‐OI variation (Figure S2). To avoid bias from admixed ancestry in downstream analyzes of diversity, demography, and selection, we excluded these 17 individuals from subsequent analyzes.

Nucleotide diversity (π) differed markedly among the three lineages, ranging from 5.25 to 9.48 × 10^−4^ (Figure S3). *O. kokonorica* (OK) exhibited the highest genetic diversity, together with the lowest LD levels and the most rapid LD decay (Figure S4), consistent with a larger effective population size and more frequent recombination. In contrast, OT showed elevated LD and substantially reduced π, likely reflecting a smaller *Ne* or stronger historical drift. Pairwise genome‐wide *F*_ST_ values ranged from 0.38 to 0.50, reflecting strong genetic differentiation among the three lineages. This deep divergence was further supported by the mean pairwise nucleotide differences (*D*_xy_) in interlineage comparisons (Table S3).

### 3.2 Demographic and Divergence History of *Orinus*

**FIGURE 3:**
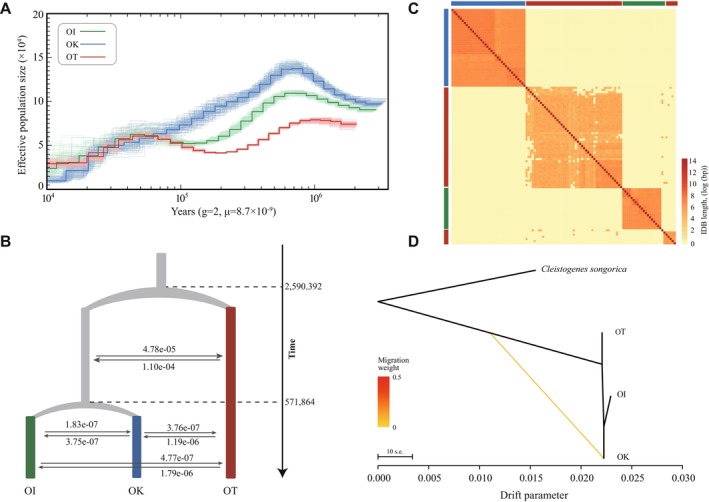
Demographic history, divergence, and gene flow among the three *Orinus* species. (A) Historical changes in effective population size (*Ne*) inferred using the PSMC model for OK, OI, and OT. Solid lines represent median estimates, and light‐colored curves denote bootstrap replicates. (B) Best‐fitting demographic model inferred from fastsimcoal2 based on pairwise joint site frequency spectra. Bidirectional migration rates (arrows) indicate persistent but asymmetric gene flow. (C) Heatmap of pairwise identity‐by‐descent (IBD) block sharing across all individuals. (D) TreeMix graph showing population relationships and historical migration. Branch lengths represent drift parameters.

To investigate the evolutionary history of the three lineages, we first reconstructed changes in effective population size (*Ne*) using the Pairwise Sequentially Markovian Coalescent (PSMC) model. All three species had relatively small *Ne* during the ancient time intervals (> 1 Mya), followed by a prolonged increase during the mid‐Pleistocene, reaching maxima around 700–800 thousand years ago (kya; Figure 3A). Thereafter, *Ne* gradually declined towards the present in all lineages. Throughout most of their history, OK maintained the largest effective population size, OI showed intermediate values, and OT had the smallest Ne. These broadly parallel demographic trajectories indicate that *Orinus* demography was strongly shaped by Pleistocene climatic oscillations.

To evaluate alternative divergence models, we analyzed pairwise joint site frequency spectra using a composite‐likelihood framework implemented in fastsimcoal2 (Figures S5–S7). The best‐fitting model supports an early divergence of OT from the common ancestor ~2.6 Mya (95% CI: 2.46–2.49 Mya), followed by the split between OK and OI ~572 kya (95% CI: 555–589 kya) (Figure 3B; Tables S4 and S5). The inferred migration parameters reveal persistent but asymmetric post‐speciation gene flow, with substantially higher historical introgression between OT and the ancestral OK‐OI lineage than between OK and OI. Patterns of isolation by distance further support these relationships: individuals cluster strongly by species in the IBD heatmap (Figure 3C), consistent with long‐term geographic isolation and restricted contemporary gene flow. TreeMix analysis recovered a similar topology (Figure 3D), with OT forming the deepest branch and showing the longest drift parameter, reflecting its smaller *Ne* and stronger drift. Together, these results indicate that the three *Orinus* species experienced prolonged divergence with heterogeneous gene flow, including stronger early gene flow involving OT and the ancestral OK‐OI lineage, followed by increasingly localized introgression during later stages of lineage formation.

### 3.3 Genomic Islands of Divergence Between Lineages

**FIGURE 4:**
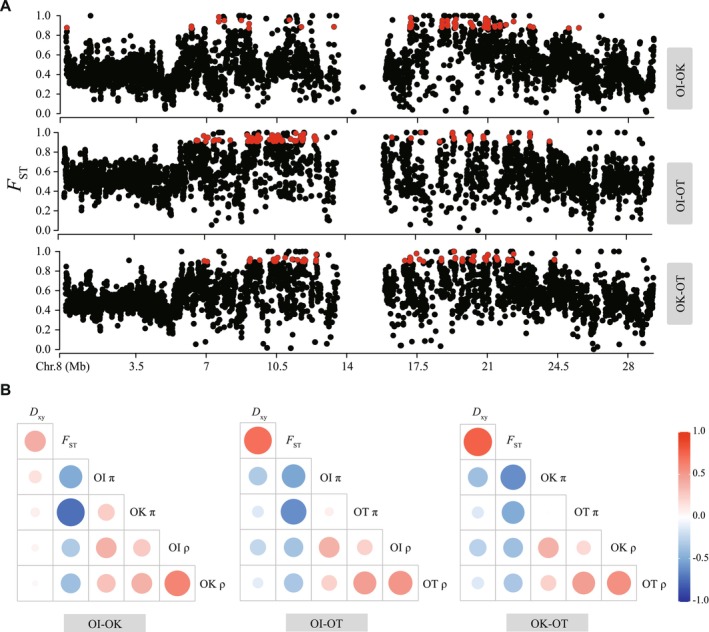
Heterogeneous genomic divergence among *Orinus* species. (A) Window‐based *F*_ST_ values along an example chromosome 8 for three pairwise species comparisons (see Figure S9 for all chromosomes). Genomic islands of divergence are shown in red. (B) Correlation matrices showing relationships among genomic parameters for each species pair. Circle size and color denote the strength and direction of correlations among *F*_ST_, *D*_xy_, nucleotide diversity (π) for each species, and recombination rate (ρ). Warm colors indicate positive correlations and cool colors indicate negative correlations.

The windowed *F*_ST_ distributions differed significantly among lineage pairs (Kolmogorov–Smirnov tests, *p* < 2.2 × 10^−16^ for all comparisons; Figure S8). The OI‐OK comparison displayed the lowest differentiation, with most windows concentrated at low *F*_ST_ values, whereas both OI‐OT and OK‐OT exhibited right‐skewed distributions with a higher proportion of highly differentiated windows. Sliding‐window genome scans further revealed strongly heterogeneous patterns of genomic divergence across all comparisons (Figure S8). The top 1% *F*_ST_ outliers comprised 2229 20‐kb windows (Table S6), with partial overlap among lineage pairs (Table S7). After merging adjacent outlier windows, we identified 1009 genomic islands of divergence that were broadly distributed across the genome but clustered in certain regions (Figures 4A and S9). Compared with non‐island regions, genomic islands showed markedly reduced nucleotide diversity (lower π), lower Tajima's *D*, increased linkage disequilibrium (higher LD), and elevated *D*_*xy*_. Gene annotation revealed that these islands collectively contained 993 genes, which likely contribute to species‐specific differentiation and local adaptation.

We further examined what factors contributed to the formation of genomic islands based on the values of *D*_xy_. We compared the level of *D*_xy_ in genomic islands to that of the genome background in each pair of lineages. We recovered significantly elevated *D*_xy_ in genomic islands for all pairwise comparisons, regardless of the rate of gene flow between lineages under comparison. This observation suggests that haplotypes that include genomic islands may have become genetically isolated before the rest of the genomes of the lineage pairs under comparison. We additionally examined changes in recombination rates and found that the population‐scale recombination rate (ρ = 4*Ne*r) was significantly reduced in genomic islands compared with the rest of the genome for all paired comparisons (Table S8). However, the correlation between recombination rates ρ and absolute divergence *D*_xy_ across the whole genome, although significant, was very low (Figure 4B).

### 3.4 Genes Under Positive Selection

Combined HKA and PBS analyzes identified 1252 unique positively selected genes (PSGs) across the three species (OT: 475; OK: 534; OI: 531), including 288 shared by at least two species (Table S9). Within genomic islands, we detected 197, 186, and 247 PSG calls in OT, OK, and OI, respectively, corresponding to 528 unique genes, of which 102 (19.3%) are shared by at least two species—a proportion significantly higher than expected by chance (*χ* ^*2*^ test, *p* < 0.001). These patterns suggest that divergent haplotypes were maintained in the ancestral population and subsequently subjected to strong, species‐specific divergent selection during speciation.

Functional annotation showed that PSGs within genomic islands were enriched for biological processes related to organ development and environmental adaptation (Table S10). Among the six drought‐responsive PSGs, three were shared by multiple species, consistent with convergent adaptation to the arid environment of the QTP, whereas the remaining three were species‐specific and likely reflect localized drought responses (Table S11). Of the four light‐responsive PSGs, two were shared between OT and OI, potentially associated with adaptation to high‐radiation alpine habitats. In addition, we identified several species‐specific PSGs with functions related to salt‐stress tolerance (e.g., *LIIP1*, *MSRA4*), flowering‐time regulation (e.g., *GHD7*, *WTR31*), DNA damage repair (*FIGL1*, *PMA5*), pollen development (*PKSB*), leaf development (*REL2*), and seed development (*MIP2*) (Table S11). These functionally diverse PSGs likely contribute to ecological differentiation and phenotypic divergence among the three species.

## 4 Discussion

### 4.1 Quaternary Climate Change Promoted Speciation With Gene Flow and Secondary Hybridization

Population structure analysis, PCA, and phylogenetic reconstruction based on genome‐wide resequencing data consistently reveal that OT, OK, and OI are genetically well differentiated, with substantial interspecific divergence (*F*_ST_ = 0.38–0.50). These values exceed those typically reported for wind‐pollinated congeners, where airborne pollen dispersal promotes extensive gene flow and consequently low differentiation (Culley et al. 2002; Kremer et al. 2012; Ma et al. 2018). In contrast, *Orinus* species are perennial grasses with plant heights ≤ 1 m, which inherently restrict pollen dispersal distance. The complex topography of the QTP likely further impedes long‐distance pollen‐mediated gene flow, reinforcing interspecific differentiation.

Historical climatic fluctuations likely contributed strongly to the divergence of *Orinus* lineages. Demographic modeling shows that *Orinus* began diversifying ~2.6 Mya, with OK and OI diverging around 0.57 Mya, both within the Quaternary. Quaternary climatic oscillations reshaped species distributions, population genetic structures, and diversification processes across the QTP (Kadereit and Abbott 2021). The plateau experienced at least four major glacial–interglacial cycles over the past ~1.2 Mya, characterized by extreme temperature drops, habitat fragmentation, and range contractions (Lehmkuhl and Owen 2005; Zheng et al. 2002). Similar patterns have been reported in QTP plant groups, including *Primula* (Ren et al. 2017) and *Medicago* (Guo et al. 2023). Our results indicate that the divergence of OT coincided with the onset of early Quaternary glaciation, whereas the split between OK and OI aligns with the Naynayxungla Glaciation (0.8–0.5 Mya), the most extensive Pleistocene glaciation on the QTP. These climatic events likely fragmented ancestral populations, severing gene flow between isolated subpopulations and promoting geographic isolation. The ancestral *Orinus* lineage likely split into a Himalayan lineage (OT progenitor) and a northern QTP lineage, with the latter further diverging into OK and OI during subsequent glacial maxima. Despite being the earliest‐diverging lineage, OT shows the lowest genetic diversity (π = 5.25 × 10^−4^). This paradox is consistent with repeated glacially driven bottlenecks: OT populations likely contracted into low‐elevation refugia during glacial periods and recolonized high elevations during interglacials. Such cycles can repeatedly reduce effective population sizes through founder effects. In contrast, OK and OI, which occupy lower elevations and broader ranges, may have persisted in multiple refugia, thereby retaining higher levels of genetic diversity.

Although OI and OK are recovered as distinct species by genome‐wide data, both taxa contain geographically localized subgroups associated with admixture. In OI, individuals from OI6‐OI7 form a minor subcluster in the NJ tree and PCA and show ca. 16% OK ancestry in ADMIXTURE; in OK, individuals from OK14‐OK16 also display mixed ancestry. Because these groups are geographically restricted and remain nested within the broader OI and OK clusters, we interpret them as phylogeographic structure combined with localized introgression, rather than as evidence for additional species‐level lineages. This pattern indicates that species divergence between OI and OK has been maintained at the genome‐wide scale, whereas incomplete reproductive isolation has allowed limited and spatially heterogeneous gene flow in localized contact zones. Comparable patterns of phylogeographic structure and introgression have also been reported in *Ostryopsis* (Liu et al. 2014), *Picea* (Sun et al. 2014), and the eastern North American white oak syngameon (Ribicoff et al. 2025). Together with the demographic modeling, these results suggest that reproductive isolation between OI and OK, and more broadly among the three *Orinus* species, is substantial but incomplete, with gene flow largely restricted to localized contact zones. The apparent allopatry of present‐day populations may reflect incomplete spatial sampling or past range shifts during climatic oscillations. Future studies with denser sampling across potential OI‐OK contact zones will be essential for clarifying the strength of reproductive barriers and the dynamics of hybridization.

After removing admixed individuals, coalescent simulations based on the site frequency spectrum revealed persistent gene flow among all species pairs following their initial divergence—supporting a speciation‐with‐gene‐flow model (Guo et al. 2023; Hu et al. 2021; Ma et al. 2018; Wu 2001). Notably, early gene flow between OT and the ancestral OK‐OI lineage was substantially higher than recent interspecific gene flow, suggesting that reproductive isolation was initially weak and intensified over time. This gradual strengthening of species boundaries was likely driven by Quaternary glacial advances, which caused sustained declines in effective population sizes, forced populations into geographically isolated refugia, and disrupted gene flow. Postglacial expansions allowed secondary contact, but by then, enhanced reproductive isolation—reinforced by divergent ecological selection in the heterogeneous environments of the QTP—maintained species boundaries, limiting hybridization to rare contact zones. As the three species occupy distinct ecological niches, environmental heterogeneity likely played a major role in counteracting gene flow and reinforcing divergence, consistent with the recognized dynamic balance between selection and gene flow in driving speciation in closely related lineages (Michel et al. 2010; Sexton et al. 2009).

### 4.2 Genomic Islands of Divergence and Ecological Adaptation

Across the genome, OT, OK, and OI exhibit highly heterogeneous patterns of genetic differentiation, with numerous *F*_ST_ defined genomic islands scattered along chromosomes, consistent with divergence under a speciation‐with‐gene‐flow scenario (Guo et al. 2023; Ma et al. 2018). As in previous studies, these genomic islands show significantly elevated *D*_xy_, reflecting deep evolutionary divergence in these regions (Hu et al. 2021, 2022). Two major mechanisms may underlie such islands: (i) differential sorting of ancestral divergent haplotypes (Han et al. 2017), and (ii) locally reduced gene flow relative to the genomic background (Feder et al. 2012; Nosil et al. 2009). However, neither historical gene flow nor divergence times were correlated with the number or size of genomic islands across the three species, suggesting that locally restricted gene flow played only a minor role in island formation. Instead, genomic islands consistently exhibited elevated LD but reduced π, Tajima's *D*, and recombination rates. Together with the significantly higher *D*_xy_ values in islands than in genomic background, these patterns are most consistent with divergent selection acting on ancestral polymorphisms, with linked sites hitchhiking to high divergence. Consistent with this interpretation, many PSGs were shared across species, implying that selection targeted ancient haplotypes segregating in their common ancestor.

Although closely related, the three species occupy distinct ecological niches and display clear morphological differentiation. OT occurs at the highest elevations (3600–4700 m) on dry sandy substrates of the northern Himalayas; OK inhabits arid slopes and alpine grasslands in Qinghai and northeastern Tibet (< 4000 m); and OI is restricted to the northern Hengduan Mountains (3300–3800 m). Morphological traits—including lemma and rachilla trichomes, pigmentation patterns, and spikelet length—correspond well with their contrasting ecological contexts (Su et al. 2015). These heterogeneous environments have likely imposed different selective pressures, contributing to lineage‐specific retention of ancestral haplotypes within genomic islands. In total, HKA and PBS analyzes identified 1252 PSGs, including 528 located within genomic islands (Table S9). Many PSGs have *Arabidopsis thaliana* orthologs associated with stress responses and organ development. Several drought‐ and light‐responsive PSGs are shared among two or all three species, consistent with convergent adaptation to the arid, high‐radiation conditions of the QTP. For example, *RHA2A*, an ABA‐signaling regulator that enhances stomatal closure and drought tolerance (Bu et al. 2009; Li et al. 2011), is under selection in all species. Beyond these shared PSGs, most are species‐specific, including salt‐stress response genes (three in OI, one in OK, none in OT) and genes involved in flowering time, DNA repair, pollen development, and leaf or seed development (Table S11). These lineage‐specific PSGs likely reflect divergent ecological regimes across the QTP and support a scenario in which distinct ancestral haplotypes were selectively retained in different species, contributing to ecological divergence, morphological differentiation, and ultimately speciation.

## Author Contributions

**Hongyin Hu:** data curation (equal), visualization (equal), writing – original draft (equal), writing – review and editing (equal). **Ai Liu:** data curation (equal), investigation (equal), project administration (equal), writing – original draft (equal). **Xue Yang:** data curation (equal), investigation (equal), project administration (equal), visualization (equal), writing – original draft (equal). **Juan Lu:** investigation (equal), methodology (equal), visualization (equal). **Kunjing Qu:** investigation (equal), methodology (equal), visualization (equal). **Jinyuan Chen:** investigation (equal), methodology (equal), visualization (equal). **Xu Su:** investigation (equal). **Guangpeng Ren:** funding acquisition (equal), investigation (equal), project administration (equal), writing – review and editing (equal).

## Funding

This work was supported by Gansu Provincial Science and Technology Major Projects (Grant 22ZD6NA007), Second Tibetan Plateau Scientific Expedition and Research (STEP) program (Grant 2019QZKK0502), Fundamental Research Funds for the Central Universities (Grant lzujbky‐2024‐ey01) and The Science and technology program of Gansu Province (Grant 25JRRA633).

## Conflicts of Interest

The authors declare no conflicts of interest.

## Supporting information


**Figure S1:** Cross‐validation (CV) error of ADMIXTURE clustering for *K* = 1–6. The CV error declines sharply from *K* = 1 to *K* = 3 and then reaches a plateau with only marginal improvement at higher *K* values. Therefore, *K* = 3 was considered the optimal number of genetic clusters and was used for the population structure analysis.
**Figure S2:** PCA based on genome‐wide SNPs. PC2 (17.1%) and PC3 (9.9%) clearly distinguish OI (green), OK (blue), and OT (red), with no overlap among clusters.
**Figure S3:** Nucleotide diversity (π) and pairwise genetic differentiation (*F*_ST_) among the three *Orinus* lineages. Each circle represents one lineage, with its nucleotide diversity (π) shown in parentheses. Dashed lines denote pairwise genome‐wide *F*_ST_ values: OT‐OI (0.46), OI‐OK (0.38), and OT‐OK (0.50).
**Figure S4:** Linkage disequilibrium (LD) decay patterns among the three *Orinus* lineages. Genome‐wide LD decay was estimated using pairwise *r* ^*2*^ values plotted against physical distance. OK shows the lowest LD and fastest decay, OI is intermediate, and OT displays the highest LD and slowest decay.
**Figure S5:** Schematic diagram of all possible topological structures of these three lineages used in fastsimcoal2 to infer demographic parameters. For each topological structure, the parameters of gene flow, divergence time, and effective population sizes were flexible, then we performed parameter estimation for 100 independent runs and chose the model with the highest likelihood. Note that the topological structure of model 3 was best supported according to the value of the likelihoods and Akaike's information criterion (AIC).
**Figure S6:** Schematic diagram of all possible topological structures of the gene flow used in fastsimcoal2. Note that the gene flow topological structure of model 12 was best supported according to the value of the likelihoods and Akaike's information criterion (AIC).
**Figure S7:** Best‐supported demographic model (M12) inferred with fastsimcoal2 for OT (*O. thoroldii*), OK (*O. kokonorica*), and OI ( *O. intermedius* ). TDIV0 and TDIV1 indicate the two divergence times. Arrows indicate gene flow, including ancestral gene flow between OT and the ancestral OK‐OI lineage and recent gene flow among extant lineages. Parameter names follow the fastsimcoal2 model specification.
**Figure S8:** *F*_ST_ distributions for each pair of lineages. The differences in the distribution shape between lineage pairs were determined by the Kolmogorov–Smirnov test. The values of the Kolmogorov–Smirnov statistic and *p*‐values were shown below and above the diagonal, respectively.
**Figure S9:** Pairwise genetic divergence (*F*_ST_) in 20‐kb sliding windows across all chromosomes for all comparisons. Genomic islands of divergence are shown in red.
**Table S1:** Overview of resequence sample information and sequencing statistics.
**Table S2:** Summary of individual‐ and site‐level missingness in the final filtered SNP dataset across the three *Orinus* species.
**Table S3:** Population genetic summary statistics. Mean values of nucleotide diversity π, Tajima's D statistic, pairwise relative measure of differentiation (*F*_ST_, below the diagonal), and absolute divergence (*D*_xy_, above the diagonal) among the three species.
**Table S4:** The AIC value for the demographic scenario modeled in fastsimcoal2.
**Table S5:** Inference parameters estimated with 95% confidence intervals for the best fitting demographic scenario modeled in fastsimcoal2, with parameters corresponding to the model shown in Figure S7.
**Table S6:** The number of outlier windows and genomic islands after combining consecutive windows for all pairwise comparisons. The number of genes and Positively Selected Genes (PSGs) within these islands is also counted.
**Table S7:** The number of shared outlier windows between paired comparisons.
**Table S8:** Comparison of population genomic parameters of genomic islands with the rest of the genomic regions for all pairwise comparisons by the Mann–Whitney *U* test.
**Table S9:** The number of PSGs between paired comparisons.
**Table S10:** Results from Gene Ontology enrichment analysis for PSGs.
**Table S11:** List of some PSGs with putative functions associated with ecological adaptation and morphological divergence in the three species.

## Data Availability

All raw sequences used in this study have been submitted to NGDC (https://ngdc.cncb.ac.cn/) under the Bioproject accession number PRJCA051823. Scripts and fastsimcoal2 model files tested are available at GitHub (https://github.com/HongyinHu/Orinus_Evolution_2026.git).
