# Supplementary material for: Genomic Insights Into Species Divergence and Adaptive Evolution in the Grass Genus Orinus on the Qinghai‐Tibet Plateau
Source: Ecol Evol. 2026 May 10;16(5):e73641. doi: 10.1002/ece3.73641 (PMC13158524; doi:10.1002/ece3.73641)
Supplement: Supplementary file 1 — Figure S1: Cross‐validation (CV) error of ADMIXTURE clustering for K = 1–6. The CV error declines sharply from K = 1 to K = 3 and then reaches a plateau with only marginal improvement at higher K values. Therefore, K = 3 was considered the optimal number of genetic clusters and was used for the population structure analysis. Figure S2: PCA based on genome‐wide SNPs. PC2 (17.1%) and PC3 (9.9%) clearly distinguish OI (green), OK (blue), and OT (red), with no overlap among clusters. Figure S3: Nucleotide diversity (π) and pairwise genetic differentiation (F ST) among the three Orinus lineages. Each circle represents one lineage, with its nucleotide diversity (π) shown in parentheses. Dashed lines denote pairwise genome‐wide F ST values: OT‐OI (0.46), OI‐OK (0.38), and OT‐OK (0.50). Figure S4: Linkage disequilibrium (LD) decay patterns among the three Orinus lineages. Genome‐wide LD decay was estimated using pairwise r 2 values plotted against physical distance. OK shows the lowest LD and fastest decay, OI is intermediate, and OT displays the highest LD and slowest decay. Figure S5: Schematic diagram of all possible topological structures of these three lineages used in fastsimcoal2 to infer demographic parameters. For each topological structure, the parameters of gene flow, divergence time, and effective population sizes were flexible, then we performed parameter estimation for 100 independent runs and chose the model with the highest likelihood. Note that the topological structure of model 3 was best supported according to the value of the likelihoods and Akaike's information criterion (AIC). Figure S6: Schematic diagram of all possible topological structures of the gene flow used in fastsimcoal2. Note that the gene flow topological structure of model 12 was best supported according to the value of the likelihoods and Akaike's information criterion (AIC). Figure S7: Best‐supported demographic model (M12) inferred with fastsimcoal2 for OT (O. thoroldii), OK (O. kokonor [file ECE3-16-e73641-s001.docx]

# Supporting Information

## Supporting Figures


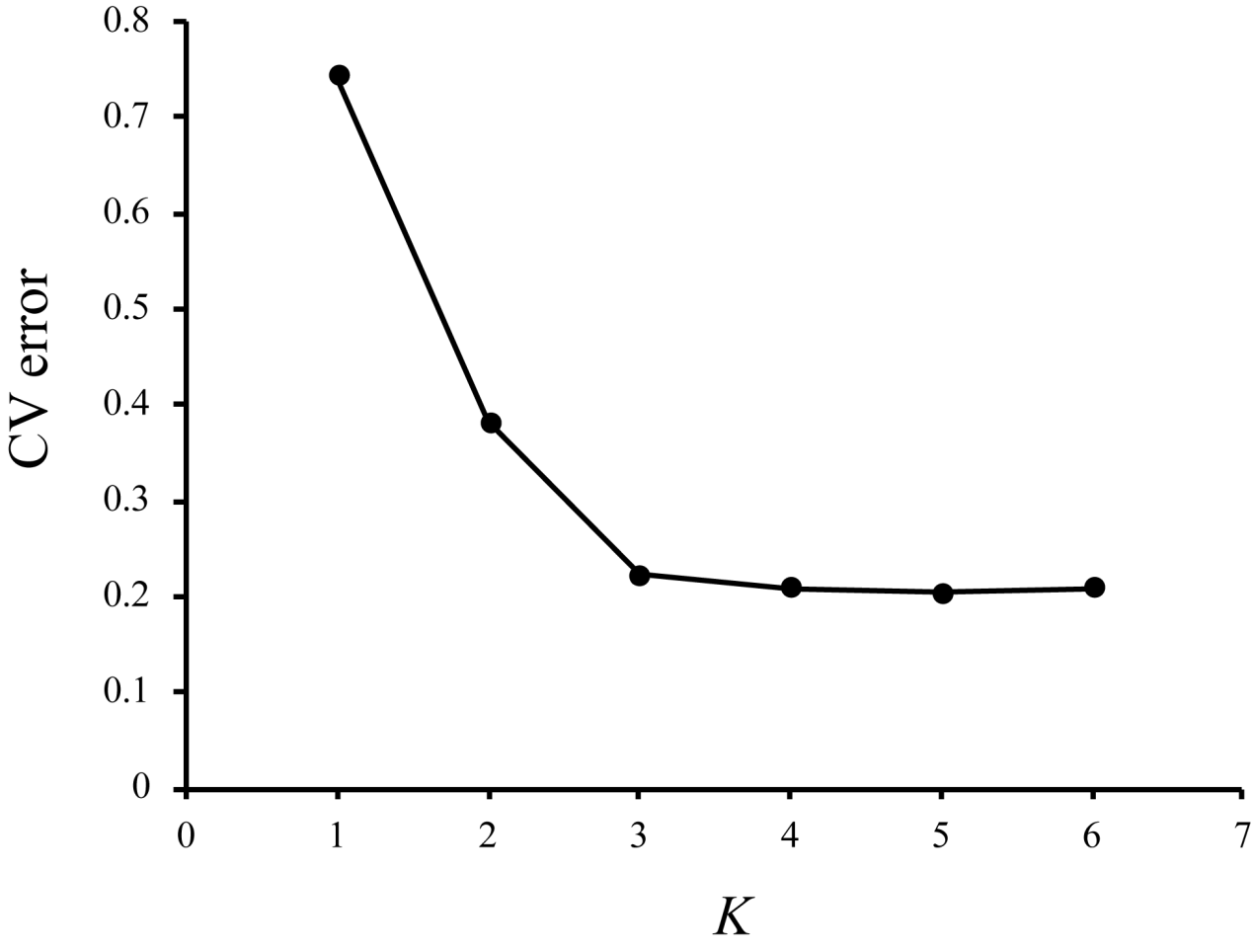


### Fig. S1. Cross-validation (CV) error of ADMIXTURE clustering for *K* = 1-6. The CV error declines sharply from *K* = 1 to *K* = 3 and then reaches a plateau with only marginal improvement at higher *K* values. Therefore, *K* = 3 was considered the optimal number of genetic clusters and was used for the population structure analysis.


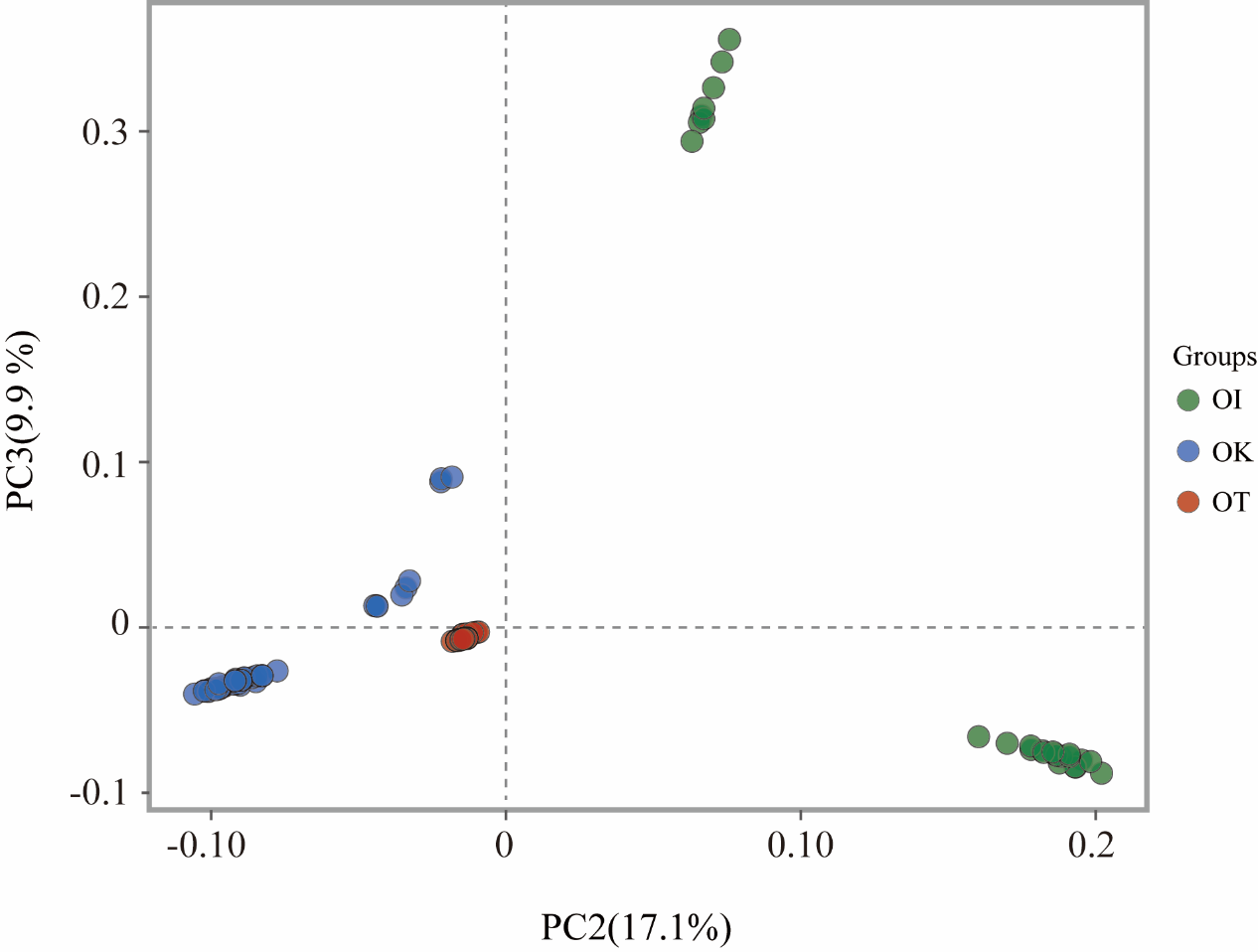


### ****Fig. S2. PCA based on genome-wide SNPs.**** PC2 (17.1%) and PC3 (9.9%) clearly distinguish OI (green), OK (blue), and OT (red), with no overlap among clusters.


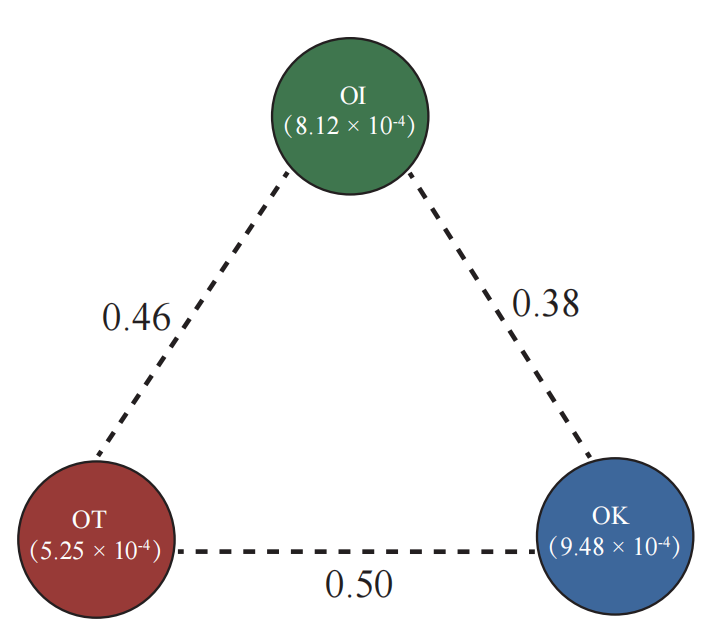


### ****Fig. S3. Nucleotide diversity (π) and pairwise genetic differentiation (*F*_ST_) among the three**** Orinus ****lineages.**** Each circle represents one lineage, with its nucleotide diversity (π) shown in parentheses. Dashed lines denote pairwise genome-wide *F*_ST_ values: OT-OI (0.46), OI-OK (0.38), and OT-OK (0.50).


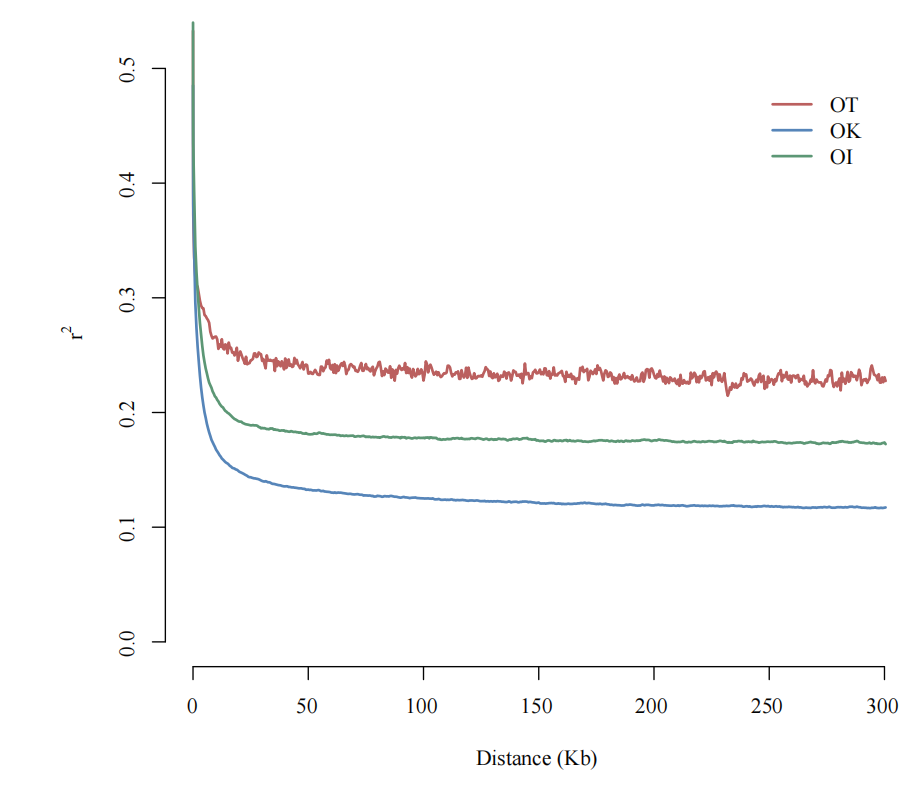


### ****Fig. S4. Linkage disequilibrium (LD) decay patterns among the three**** Orinus ****lineages.**** Genome-wide LD decay was estimated using pairwise *r²* values plotted against physical distance. OK shows the lowest LD and fastest decay, OI is intermediate, and OT displays the highest LD and slowest decay.


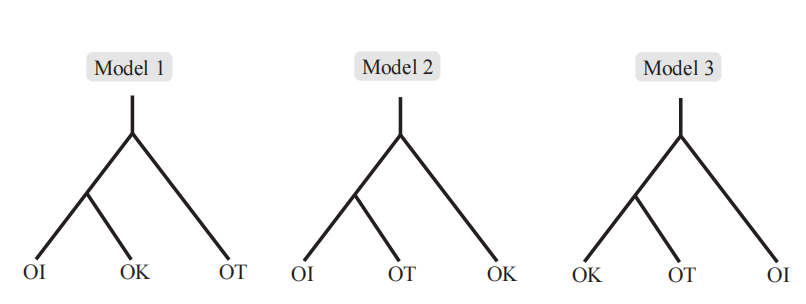


### Fig. S5. Schematic diagram of all possible topological structures of these three lineages used in fastsimcoal2 to infer demographic parameters. For each topological structure, the parameters of gene flow, divergence time, and effective population sizes were flexible, then we performed parameter estimation for 100 independent runs and chose the model with the highest likelihood. Note that the topological structure of model 3 was best supported according to the value of the likelihoods and Akaike’s information criterion (AIC).


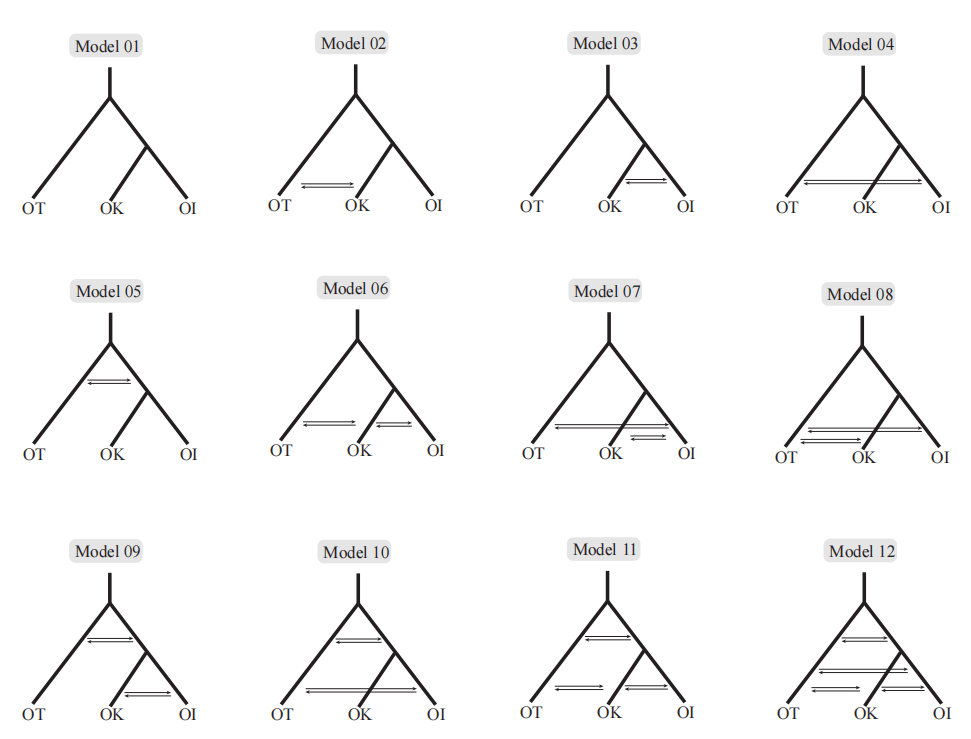


### Fig. S6. Schematic diagram of all possible topological structures of the gene flow used in fastsimcoal2. Note that the gene flow topological structure of model 12 was best supported according to the value of the likelihoods and Akaike’s information criterion (AIC).


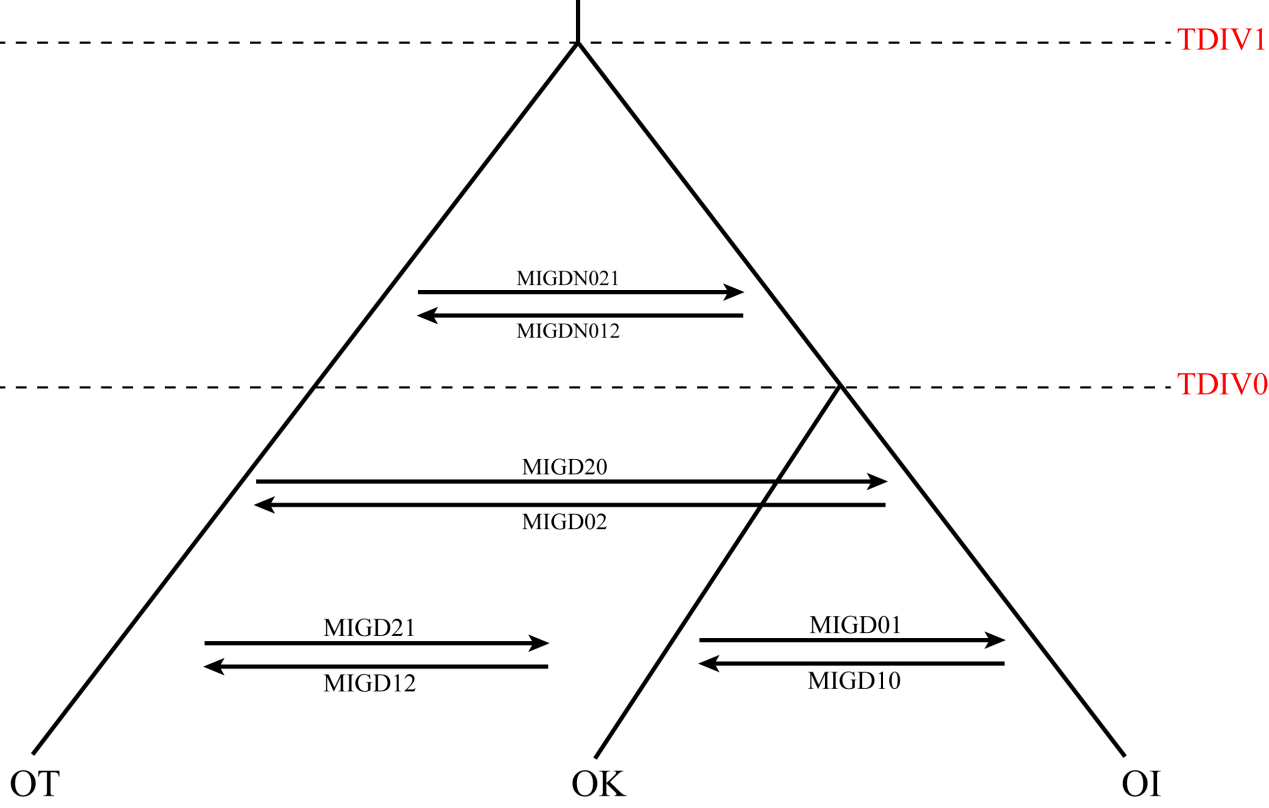


### Fig. S7. Best-supported demographic model (M12) inferred with fastsimcoal2 for OT (*O. thoroldii*), OK (*O. kokonorica*), and OI (*O. intermedius*). TDIV0 and TDIV1 indicate the two divergence times. Arrows indicate gene flow, including ancestral gene flow between OT and the ancestral OK-OI lineage and recent gene flow among extant lineages. Parameter names follow the fastsimcoal2 model specification.


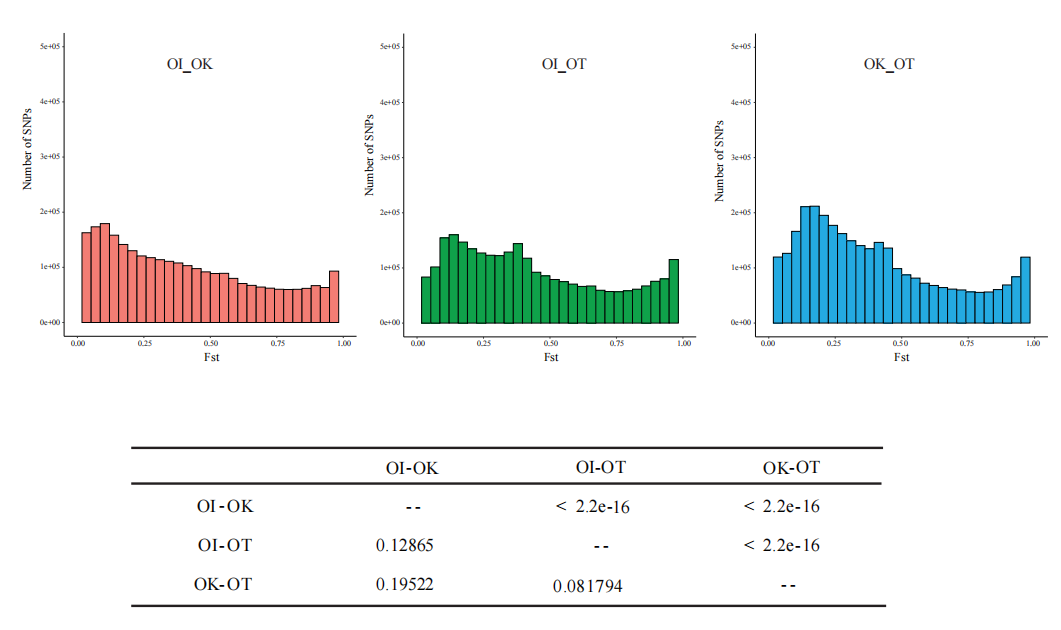


### Fig. S8. *F*_ST_ distributions for each pair of lineages. The differences in the distribution shape between lineage pairs were determined by the Kolmogorov-Smirnov test. The values of the Kolmogorov-Smirnov statistic and *P*-values were shown below and above the diagonal, respectively.


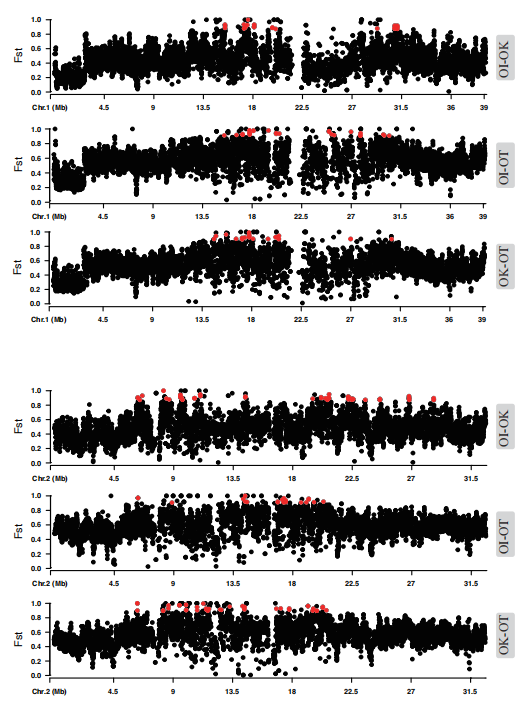

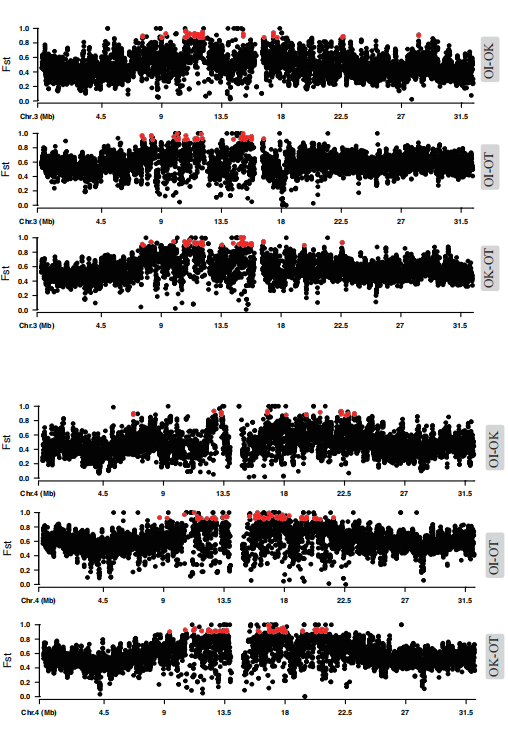

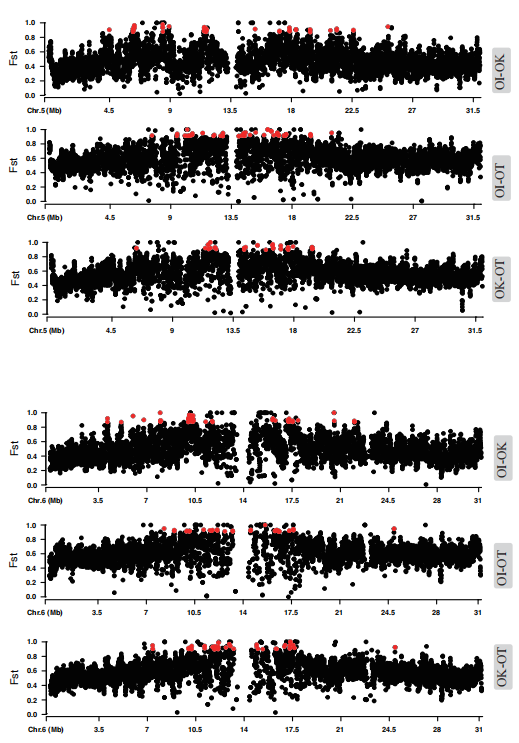

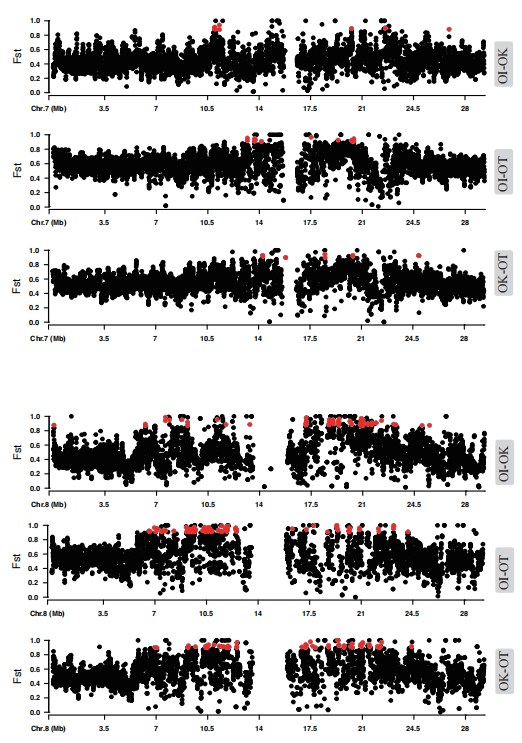

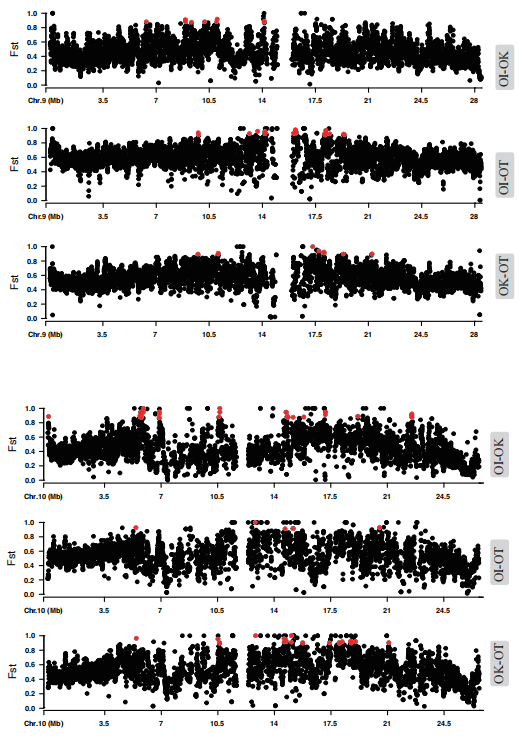

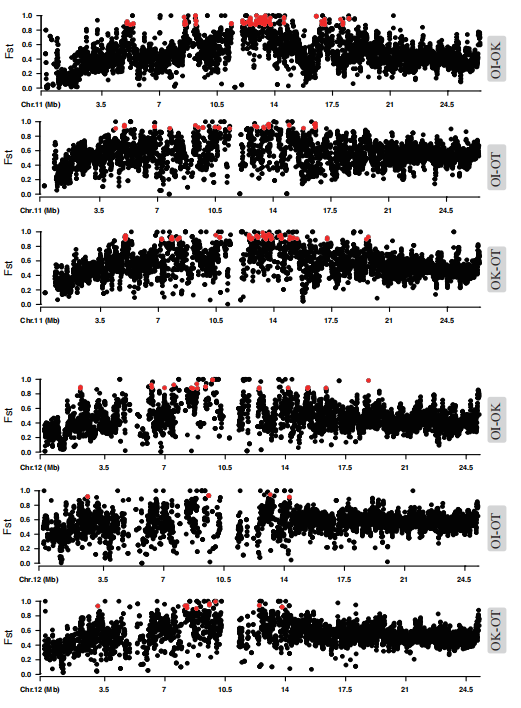

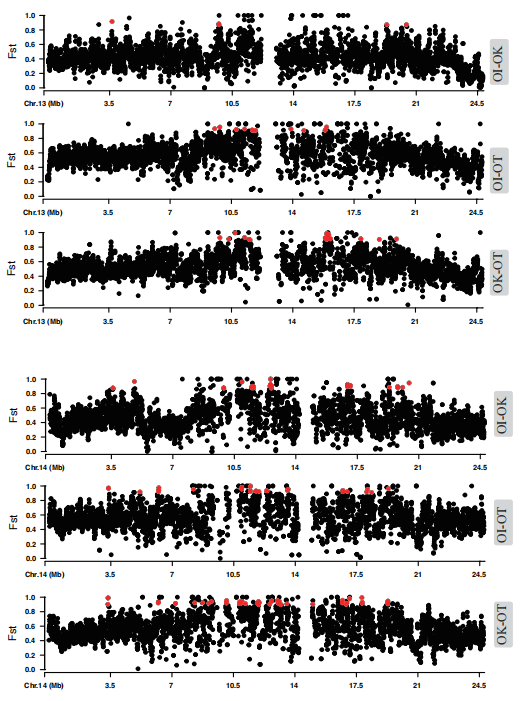

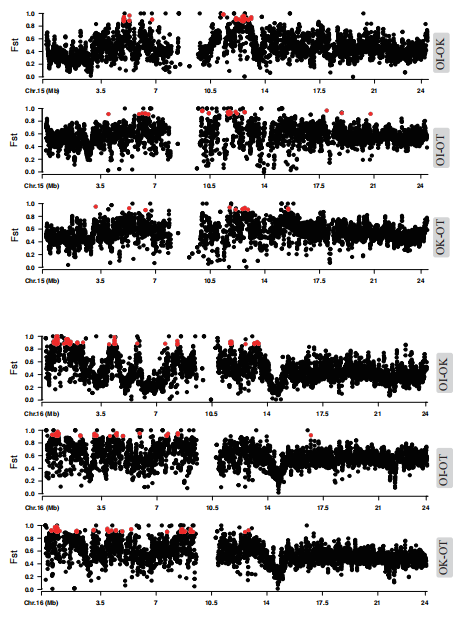

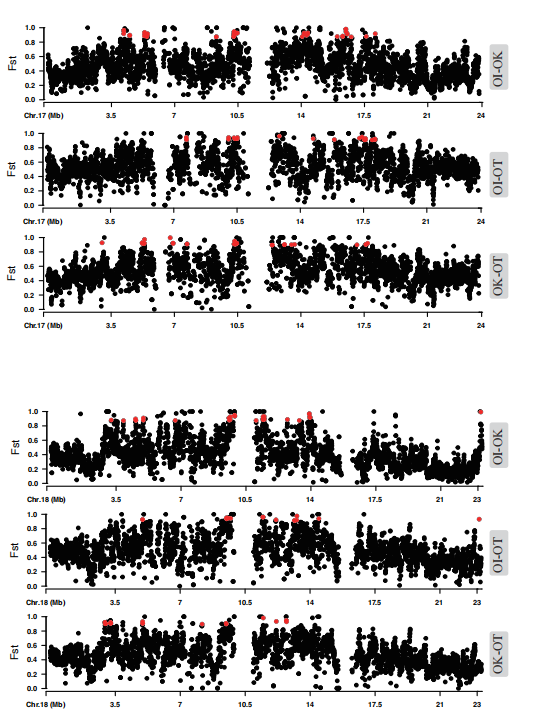

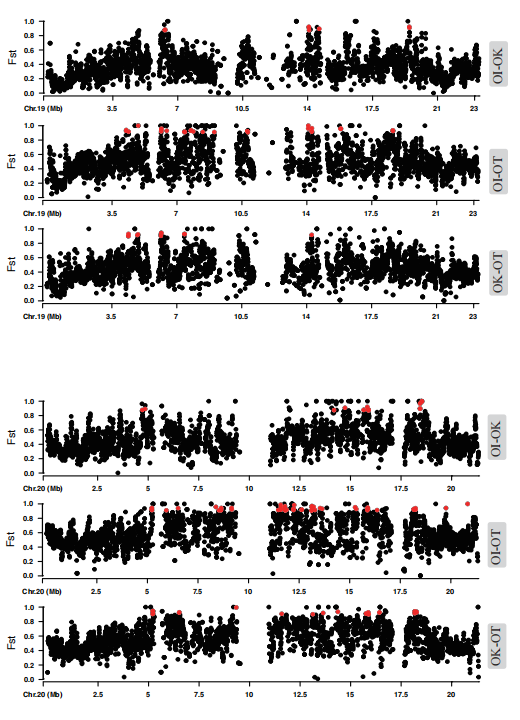


### Fig. S9. Pairwise genetic divergence (*F*_ST_) in 20-kb sliding windows across all chromosomes for all comparisons. Genomic islands of divergence are shown in red.

## Supporting Tables

### Table S1. Overview of resequence sample information and sequencing statistics

| **Sample Id** | **Latitude/N** | **Longitude/E** | **Altitude (m)** | **Region** | **Map Ratio** | **Clean Read (bp)** | **Genome Coverage^1^** | **Effective Depth^2^** |
| --- | --- | --- | --- | --- | --- | --- | --- | --- |
| OI1_1 | 32.913 | 101.783 | 3379 | Aba, Sichuan | 92.15% | 8,772,638,086 | 80.08% | 15.78 |
| OI1_2 | 32.913 | 101.783 | 3379 | Aba, Sichuan | 97.86% | 9,774,414,109 | 80.58% | 17.58 |
| OI1_3 | 32.913 | 101.783 | 3379 | Aba, Sichuan | 98.05% | 9,399,549,509 | 80.66% | 16.91 |
| OI1_4 | 32.913 | 101.783 | 3379 | Aba, Sichuan | 98.15% | 10,330,530,250 | 80.97% | 18.58 |
| OI2_1 | 30.622 | 101.404 | 3573 | Daofu, Sichuan | 96.50% | 9,862,779,439 | 80.61% | 17.74 |
| OI2_2 | 30.622 | 101.404 | 3573 | Daofu, Sichuan | 98.17% | 10,462,025,768 | 80.79% | 18.82 |
| OI2_3 | 30.622 | 101.404 | 3573 | Daofu, Sichuan | 97.69% | 9,896,259,378 | 80.32% | 17.80 |
| OI2_4 | 30.622 | 101.404 | 3573 | Daofu, Sichuan | 98.39% | 9,139,294,196 | 79.91% | 16.44 |
| OI3_1 | 31.771 | 100.983 | 3478 | Rangtang, Sichuan | 96.10% | 10,001,731,495 | 80.79% | 17.99 |
| OI3_2 | 31.771 | 100.983 | 3478 | Rangtang, Sichuan | 98.06% | 9,328,635,481 | 80.67% | 16.78 |
| OI3_3 | 31.771 | 100.983 | 3478 | Rangtang, Sichuan | 98.17% | 10,044,107,699 | 80.84% | 18.07 |
| OI4_1 | 33.025 | 100.698 | 3852 | Banma, Qinghai | 97.96% | 9,766,676,169 | 80.81% | 17.57 |
| OI4_2 | 33.025 | 100.698 | 3852 | Banma, Qinghai | 97.82% | 10,134,423,816 | 80.85% | 18.23 |
| OI4_3 | 33.025 | 100.698 | 3852 | Banma, Qinghai | 97.63% | 9,989,719,958 | 80.78% | 17.97 |
| OI4_4 | 33.025 | 100.698 | 3852 | Banma, Qinghai | 97.97% | 9,549,339,842 | 80.71% | 17.18 |
| OI5_1 | 31.643 | 100.288 | 3534 | Luhuo, Sichuan | 98.25% | 10,126,478,168 | 81.28% | 18.21 |
| OI5_2 | 31.643 | 100.288 | 3534 | Luhuo, Sichuan | 97.96% | 10,360,804,745 | 81.31% | 18.64 |
| OI5_3 | 31.643 | 100.288 | 3534 | Luhuo, Sichuan | 97.94% | 9,225,278,845 | 81.05% | 16.59 |
| OI5_4 | 31.643 | 100.288 | 3534 | Luhuo, Sichuan | 96.95% | 10,221,448,642 | 81.48% | 18.39 |
| OI6_1 | 29.541 | 98.255 | 3522 | Mangkang, Xizang | 98.33% | 10,527,168,992 | 82.24% | 18.94 |
| OI6_2 | 29.541 | 98.255 | 3522 | Mangkang, Xizang | 98.19% | 9,678,483,905 | 82.14% | 17.41 |
| OI6_3 | 29.541 | 98.255 | 3522 | Mangkang, Xizang | 98.09% | 9,758,797,855 | 81.74% | 17.55 |
| OI6_4 | 29.541 | 98.255 | 3522 | Mangkang, Xizang | 98.29% | 9,999,451,314 | 81.95% | 17.99 |
| OI7_1 | 29.541 | 98.251 | 3507 | Mangkang, Xizang | 98.47% | 9,909,255,456 | 82.85% | 17.82 |
| OI7_2 | 29.541 | 98.251 | 3507 | Mangkang, Xizang | 98.19% | 9,289,259,132 | 81.56% | 16.71 |
| OI7_3 | 29.541 | 98.251 | 3507 | Mangkang, Xizang | 97.52% | 12,132,461,871 | 82.18% | 21.82 |
| OI7_4 | 29.541 | 98.251 | 3507 | Mangkang, Xizang | 98.21% | 9,694,118,367 | 81.50% | 17.44 |
| OK01_1 | 35.186 | 102.517 | 3007 | Xiahe, Gansu | 97.91% | 10,918,421,008 | 90.06% | 19.64 |
| OK01_2 | 35.186 | 102.517 | 3007 | Xiahe, Gansu | 94.18% | 10,180,402,176 | 90.17% | 18.31 |
| OK01_3 | 35.186 | 102.517 | 3007 | Xiahe, Gansu | 96.39% | 11,160,113,900 | 90.10% | 20.07 |
| OK02_1 | 36.62 | 101.742 | 2468 | Xining, Qinghai | 98.96% | 10,273,493,590 | 90.59% | 18.48 |
| OK02_2 | 36.62 | 101.742 | 2468 | Xining, Qinghai | 94.77% | 9,442,492,639 | 90.14% | 16.98 |
| OK02_3 | 36.62 | 101.742 | 2468 | Xining, Qinghai | 95.68% | 9,173,646,226 | 89.98% | 16.50 |
| OK04_1 | 36.184 | 100.988 | 2826 | Gonghe, Qinghai | 98.97% | 10,226,904,182 | 91.01% | 18.40 |
| OK04_2 | 36.184 | 100.988 | 2826 | Gonghe, Qinghai | 98.44% | 10,551,696,882 | 90.81% | 18.98 |
| OK04_3 | 36.184 | 100.988 | 2826 | Gonghe, Qinghai | 98.40% | 10,231,976,990 | 90.91% | 18.40 |
| OK05_1 | 36.836 | 100.835 | 3305 | Haiyan, Qinghai | 96.72% | 10,213,290,730 | 90.28% | 18.37 |
| OK05_2 | 36.836 | 100.835 | 3305 | Haiyan, Qinghai | 96.33% | 10,615,575,323 | 90.52% | 19.09 |
| OK05_3 | 36.836 | 100.835 | 3305 | Haiyan, Qinghai | 98.77% | 9,671,594,673 | 90.43% | 17.40 |
| OK06_1 | 36.1 | 100.404 | 2988 | Gonghe, Qinghai | 98.29% | 11,276,582,563 | 90.90% | 20.28 |
| OK06_2 | 36.1 | 100.404 | 2988 | Gonghe, Qinghai | 98.15% | 10,186,165,039 | 90.43% | 18.32 |
| OK06_3 | 36.1 | 100.404 | 2988 | Gonghe, Qinghai | 97.97% | 10,151,645,552 | 90.73% | 18.26 |
| OK07_1 | 36.039 | 100.315 | 3072 | Gonghe, Qinghai | 98.92% | 10,197,729,744 | 90.13% | 18.34 |
| OK07_2 | 36.039 | 100.315 | 3072 | Gonghe, Qinghai | 98.81% | 9,923,856,462 | 90.79% | 17.85 |
| OK07_3 | 36.039 | 100.315 | 3072 | Gonghe, Qinghai | 98.93% | 9,771,566,288 | 91.25% | 17.58 |
| OK08_1 | 35.703 | 100.261 | 2956 | Xinghai, Qinghai | 98.66% | 10,240,951,885 | 90.49% | 18.42 |
| OK08_2 | 35.703 | 100.261 | 2956 | Xinghai, Qinghai | 98.89% | 10,638,593,669 | 91.23% | 19.14 |
| OK08_3 | 35.703 | 100.261 | 2956 | Xinghai, Qinghai | 98.29% | 10,594,410,430 | 90.46% | 19.06 |
| OK09_1 | 31.339 | 98.134 | 3818 | Jiangda, Xizang | 98.60% | 9,483,450,525 | 89.86% | 17.06 |
| OK09_2 | 31.339 | 98.134 | 3818 | Jiangda, Xizang | 95.09% | 9,366,261,408 | 89.43% | 16.85 |
| OK09_3 | 31.339 | 98.134 | 3818 | Jiangda, Xizang | 98.25% | 9,666,687,838 | 89.49% | 17.39 |
| OK10_1 | 32.982 | 97.238 | 3493 | Yushu, Qinghai | 94.47% | 10,088,262,790 | 90.17% | 18.15 |
| OK10_2 | 32.982 | 97.238 | 3493 | Yushu, Qinghai | 98.26% | 12,100,711,836 | 90.76% | 21.77 |
| OK10_3 | 32.982 | 97.238 | 3493 | Yushu, Qinghai | 98.07% | 10,087,134,952 | 89.96% | 18.14 |
| OK11_1 | 32.49 | 96.269 | 3728 | Nangqian, Qinghai | 98.56% | 10,041,961,237 | 90.20% | 18.06 |
| OK11_2 | 32.49 | 96.269 | 3728 | Nangqian, Qinghai | 99.00% | 10,147,061,703 | 89.36% | 18.25 |
| OK11_3 | 32.49 | 96.269 | 3728 | Nangqian, Qinghai | 98.93% | 10,123,334,725 | 90.35% | 18.21 |
| OK12_1 | 32.547 | 96.196 | 4119 | Nangqian, Qinghai | 98.87% | 9,418,806,126 | 89.44% | 16.94 |
| OK12_2 | 32.547 | 96.196 | 4119 | Nangqian, Qinghai | 98.82% | 9,862,311,177 | 89.75% | 17.74 |
| OK12_3 | 32.547 | 96.196 | 4119 | Nangqian, Qinghai | 98.75% | 10,115,999,111 | 89.91% | 18.20 |
| OK13_1 | 32.547 | 96.196 | 4119 | Nangqian, Qinghai | 98.93% | 9,489,328,752 | 89.98% | 17.07 |
| OK13_2 | 32.547 | 96.196 | 4119 | Nangqian, Qinghai | 98.80% | 10,234,570,160 | 90.02% | 18.41 |
| OK13_3 | 32.547 | 96.196 | 4119 | Nangqian, Qinghai | 98.70% | 10,338,302,901 | 89.85% | 18.60 |
| OK14_1 | 30.767 | 95.574 | 3762 | Luolong, Xizang | 98.07% | 10,300,980,123 | 90.68% | 18.53 |
| OK14_2 | 30.767 | 95.574 | 3762 | Luolong, Xizang | 98.30% | 10,846,285,087 | 90.87% | 19.51 |
| OK14_3 | 30.767 | 95.574 | 3762 | Luolong, Xizang | 97.32% | 9,627,123,052 | 90.53% | 17.32 |
| OK15_1 | 30.978 | 94.726 | 3597 | Bianba, Xizang | 97.92% | 10,191,609,653 | 90.03% | 18.33 |
| OK15_2 | 30.978 | 94.726 | 3597 | Bianba, Xizang | 97.31% | 9,416,528,332 | 90.83% | 16.94 |
| OK15_3 | 30.978 | 94.726 | 3597 | Bianba, Xizang | 97.40% | 9,644,134,694 | 90.61% | 17.35 |
| OK16_1 | 31.519 | 93.533 | 3991 | Biru, Xizang | 98.07% | 9,717,179,558 | 91.08% | 17.48 |
| OK16_2 | 31.519 | 93.533 | 3991 | Biru, Xizang | 98.19% | 9,796,091,773 | 91.24% | 17.62 |
| OK16_3 | 31.519 | 93.533 | 3991 | Biru, Xizang | 98.21% | 9,860,293,058 | 91.08% | 17.74 |
| OT01_1 | 29.282 | 91.013 | 3648 | Gongga, Xizang | 96.86% | 8,797,347,087 | 72.40% | 15.82 |
| OT01_2 | 29.282 | 91.013 | 3648 | Gongga, Xizang | 95.08% | 8,673,238,810 | 72.25% | 15.60 |
| OT01_3 | 29.282 | 91.013 | 3648 | Gongga, Xizang | 96.23% | 10,578,431,135 | 73.36% | 19.03 |
| OT02_1 | 29.496 | 90.937 | 3617 | Qushui, Xizang | 96.83% | 10,818,207,081 | 73.40% | 19.46 |
| OT02_2 | 29.496 | 90.937 | 3617 | Qushui, Xizang | 96.65% | 9,712,282,889 | 73.16% | 17.47 |
| OT02_3 | 29.496 | 90.937 | 3617 | Qushui, Xizang | 96.90% | 9,145,096,748 | 72.08% | 16.45 |
| OT03_1 | 28.556 | 89.684 | 4412 | Kangma, Xizang | 96.62% | 9,040,932,848 | 71.81% | 16.26 |
| OT03_2 | 28.556 | 89.684 | 4412 | Kangma, Xizang | 96.81% | 9,263,398,561 | 72.28% | 16.66 |
| OT03_3 | 28.556 | 89.684 | 4412 | Kangma, Xizang | 96.37% | 9,046,382,604 | 72.33% | 16.27 |
| OT04_1 | 29.339 | 89.261 | 3796 | Rikaze, Xizang | 96.75% | 10,101,377,142 | 72.44% | 18.17 |
| OT04_2 | 29.339 | 89.261 | 3796 | Rikaze, Xizang | 96.83% | 9,414,722,484 | 72.31% | 16.93 |
| OT04_3 | 29.339 | 89.261 | 3796 | Rikaze, Xizang | 96.66% | 9,029,149,427 | 72.26% | 16.24 |
| OT05_1 | 29.158 | 88.171 | 4060 | Lazi, Xizang | 96.90% | 9,751,387,726 | 72.46% | 17.54 |
| OT05_2 | 29.158 | 88.171 | 4060 | Lazi, Xizang | 96.93% | 9,158,334,456 | 71.88% | 16.47 |
| OT05_3 | 29.158 | 88.171 | 4060 | Lazi, Xizang | 97.00% | 9,424,547,867 | 72.46% | 16.95 |
| OT06_1 | 28.364 | 87.766 | 4324 | Dingjie, Xizang | 96.60% | 9,417,318,057 | 71.93% | 16.94 |
| OT06_2 | 28.364 | 87.766 | 4324 | Dingjie, Xizang | 96.92% | 9,688,985,514 | 72.08% | 17.43 |
| OT06_3 | 28.364 | 87.766 | 4324 | Dingjie, Xizang | 96.65% | 9,442,563,580 | 71.94% | 16.98 |
| OT07_1 | 28.66 | 87.129 | 3852 | Dingri, Xizang | 96.93% | 9,200,327,079 | 71.94% | 16.55 |
| OT07_2 | 28.66 | 87.129 | 3852 | Dingri, Xizang | 97.02% | 9,423,925,431 | 71.85% | 16.95 |
| OT07_3 | 28.66 | 87.129 | 3852 | Dingri, Xizang | 96.82% | 9,020,507,864 | 72.09% | 16.23 |
| OT08_1 | 28.66 | 87.129 | 3852 | Dingri, Xizang | 96.89% | 9,335,184,773 | 72.17% | 16.79 |
| OT08_2 | 28.66 | 87.129 | 3852 | Dingri, Xizang | 96.79% | 9,317,398,455 | 71.86% | 16.76 |
| OT08_3 | 28.66 | 87.129 | 3852 | Dingri, Xizang | 96.75% | 9,130,075,983 | 71.54% | 16.42 |
| OT09_1 | 29.44 | 86.665 | 4593 | Angren, Xizang | 96.65% | 9,301,640,310 | 71.58% | 16.73 |
| OT09_2 | 29.44 | 86.665 | 4593 | Angren, Xizang | 94.54% | 9,151,454,334 | 72.17% | 16.46 |
| OT09_3 | 29.44 | 86.665 | 4593 | Angren, Xizang | 96.93% | 8,810,063,475 | 71.61% | 15.85 |
| OT10_1 | 28.768 | 85.537 | 4614 | Jilong, Xizang | 96.68% | 9,402,748,524 | 71.57% | 16.91 |
| OT10_2 | 28.768 | 85.537 | 4614 | Jilong, Xizang | 96.45% | 9,206,999,454 | 71.90% | 16.56 |
| OT10_3 | 28.768 | 85.537 | 4614 | Jilong, Xizang | 95.93% | 9,057,289,258 | 71.55% | 16.29 |
| OT11_1 | 29.392 | 85.516 | 4677 | Shaga, Xizang | 96.93% | 9,537,844,288 | 71.84% | 17.16 |
| OT11_2 | 29.392 | 85.516 | 4677 | Shaga, Xizang | 96.79% | 8,980,766,928 | 71.88% | 16.15 |
| OT11_3 | 29.392 | 85.516 | 4677 | Shaga, Xizang | 96.89% | 9,358,652,706 | 71.68% | 16.83 |
| OT12_1 | 29.686 | 84.147 | 4563 | Zhongba, Xizang | 96.70% | 9,166,723,473 | 71.55% | 16.49 |
| OT12_2 | 29.686 | 84.147 | 4563 | Zhongba, Xizang | 96.17% | 9,137,438,388 | 71.73% | 16.44 |
| OT12_3 | 29.686 | 84.147 | 4563 | Zhongba, Xizang | 96.57% | 8,857,445,874 | 71.27% | 15.93 |
| OT13_1 | 29.996 | 83.529 | 4582 | Zhongba, Xizang | 96.94% | 9,185,115,324 | 71.36% | 16.52 |
| OT13_2 | 29.996 | 83.529 | 4582 | Zhongba, Xizang | 96.82% | 9,227,245,028 | 71.84% | 16.60 |
| OT13_3 | 29.996 | 83.529 | 4582 | Zhongba, Xizang | 96.40% | 9,406,659,624 | 71.86% | 16.92 |
| OT14_1 | 30.81 | 81.573 | 4610 | Pulan, Xizang | 96.70% | 9,447,336,923 | 71.47% | 16.99 |
| OT14_2 | 30.81 | 81.573 | 4610 | Pulan, Xizang | 95.99% | 8,921,242,944 | 71.16% | 16.05 |
| OT14_3 | 30.81 | 81.573 | 4610 | Pulan, Xizang | 96.93% | 9,356,504,388 | 71.13% | 16.83 |
| OT15_1 | 30.366 | 81.152 | 4260 | Pulan, Xizang | 96.27% | 9,323,560,958 | 71.61% | 16.77 |
| OT15_2 | 30.366 | 81.152 | 4260 | Pulan, Xizang | 96.67% | 9,409,292,954 | 71.76% | 16.92 |
| OT15_3 | 30.366 | 81.152 | 4260 | Pulan, Xizang | 96.76% | 9,250,614,653 | 71.81% | 16.64 |
| OT16_1 | 31.179 | 80.757 | 4427 | Pulan, Xizang | 96.88% | 8,975,144,287 | 71.48% | 16.14 |
| OT16_2 | 31.179 | 80.757 | 4427 | Pulan, Xizang | 96.03% | 9,018,359,760 | 71.60% | 16.22 |
| OT16_3 | 31.179 | 80.757 | 4427 | Pulan, Xizang | 96.89% | 9,118,683,187 | 71.37% | 16.40 |
| OT17_1 | 32.572 | 80.053 | 4451 | Ali, Xizang | 97.08% | 9,171,249,291 | 71.64% | 16.50 |
| OT17_2 | 32.572 | 80.053 | 4451 | Ali, Xizang | 96.87% | 9,205,551,092 | 71.47% | 16.56 |
| OT17_3 | 32.572 | 80.053 | 4451 | Ali, Xizang | 96.78% | 9,373,128,304 | 71.65% | 16.86 |
| OT18_1 | 31.479 | 79.803 | 4434 | Zhada, Xizang | 96.12% | 9,407,143,350 | 71.70% | 16.92 |
| OT18_2 | 31.479 | 79.803 | 4434 | Zhada, Xizang | 96.80% | 9,343,548,674 | 71.63% | 16.81 |
| OT18_3 | 31.479 | 79.803 | 4434 | Zhada, Xizang | 96.70% | 9,115,497,051 | 71.41% | 16.40 |

^1^Genome coverage = Covered site length / Genome size.

^2^Effective depth = Mapped bases number / Genome size.

### Table S2. Summary of individual- and site-level missingness in the final filtered SNP dataset across the three *Orinus* species.

| **Species** | **N_individuals** | **Mean individual F_MISS** | **Median individual F_MISS** | **Mean site F_MISS** |
| --- | --- | --- | --- | --- |
| OT | 54 | 16.68% | 16.31% | 18.02% |
| OI | 27 | 12.01% | 11.90% | 6.48% |
| OK | 45 | 12.64% | 12.27% | 11.38% |

### Table S3. Population genetic summary statistics. Mean values of nucleotide diversity π, Tajima’s D statistic, pairwise relative measure of differentiation (*F*_ST_, below the diagonal), and absolute divergence (*D*_xy_, above the diagonal) among the three species.

| **Lineage** | **Number of individuals** | **π （×10 ^-4^）** | **Tajima’s D** | ***D*_xy_/*F*_ST_ (fixed SNPs)** | | |
| --- | --- | --- | --- | --- | --- | --- |
|  |  |  |  | **OT** | **OK** | **OI** |
| **OT** | 54 | 5.25 | 2.66 | -- | 0.51 | 0.48 |
| **OK** | 45 | 9.48 | 1.64 | 0.50 | -- | 0.35 |
| **OI** | 27 | 8.12 | 1.85 | 0.46 | 0.38 | -- |

### Table S4. The AIC value for the demographic scenario modeled in fastsimcoal2.

| **Model** | **AIC** |
| --- | --- |
| M01 | 12317744 |
| M02 | 10520817 |
| M03 | 12062703 |
| M04 | 11143977 |
| M05 | 11013083 |
| M06 | 10220651 |
| M07 | 10056453 |
| M08 | 9720547 |
| M09 | 10786575 |
| M10 | 10677192 |
| M11 | 10015925 |
| M12 | 9641196 |

### Table S5. Inference parameters estimated with 95% confidence intervals for the best fitting demographic scenario modeled in fastsimcoal2, with parameters corresponding to the model shown in Fig. S7.

| **Parameter** | **Point-estimate** | **95% confidence intervals** | |
| --- | --- | --- | --- |
|  |  | **Lower bound** | **Upper bound** |
| MIGDN012 | 4.78E-05 | 4.59E-05 | 4.92E-05 |
| MIGDN021 | 1.10E-04 | 1.05E-04 | 1.12E-04 |
| MIGD01 | 1.83E-07 | 1.76E-07 | 1.92E-07 |
| MIGD10 | 3.75E-07 | 3.64E-07 | 3.90E-07 |
| MIGD12 | 3.76E-07 | 3.57E-07 | 3.83E-07 |
| MIGD21 | 1.19E-06 | 1.13E-06 | 1.23E-06 |
| MIGD02 | 4.77E-07 | 4.53E-07 | 4.87E-07 |
| MIGD20 | 1.79E-06 | 1.72E-06 | 1.86E-06 |
| TDIV0 | 571,864 | 554,708 | 589,020 |
| TDIV1 | 2,590,392 | 2,460,872 | 2,694,008 |

### Table S6. The number of outlier windows and genomic islands after combining consecutive windows for all pairwise comparisons. The number of genes and Positively Selected Genes (PSGs) within these islands is also counted.

| **Pops** | **# of outlier windows** | **# of genomic islands** | **# of genes in these islands** | **# of PSGs in these islands** |
| --- | --- | --- | --- | --- |
| **OI_OK** | 905 | 324 | 788 | 247 |
| **OI_OT** | 867 | 377 | 654 | 197 |
| **OK_OT** | 889 | 381 | 627 | 186 |
| **Total** | 2229 | 1009 | 993 | 528 |

### Table S7. The number of shared outlier windows between paired comparisons.

|  | **OI_OK** | **OI_OT** | **OK_OT** |
| --- | --- | --- | --- |
| **OI_OK** | -- | -- | -- |
| **OI_OT** | 85 | -- | -- |
| **OK_OT** | 113 | 282 | -- |

### Table S8. Comparison of population genomic parameters of genomic islands with the rest of the genomic regions for all pairwise comparisons by the Mann-Whitney U test.

| **Parameter** | **Lineage** | **Genomic background** | **Genomic islands** | ***P*-value** |
| --- | --- | --- | --- | --- |
| OI_OK | | | | |
| *F*_ST_ |  | 0.40 | 0.87 | 2.20E-16 |
| *D*xy |  | 0.31 | 0.52 | 2.20E-16 |
| π | OI | 8.12×10^-4^ | 5.16×10^-5^ | 2.20E-16 |
|  | OK | 9.48×10^-4^ | 9.01×10^-5^ | 2.20E-16 |
| Tajima's *D* | OI | 1.85 | 1.12 | 0.006899 |
|  | OK | 1.64 | 0.81 | 0.001034 |
| LD (*r^2^*) | OI | 0.18 | 0.46 | 0.107 |
|  | OK | 0.13 | 0.39 | 5.26E-05 |
| *ρ* | OI | 25.81 | 7.11 | 6.13E-04 |
|  | OK | 32.92 | 6.02 | 1.46E-04 |
| OI_OT | | | | |
| *F*_ST_ |  | 0.45 | 0.80 | 2.20E-16 |
| *D*xy |  | 0.45 | 0.66 | 2.20E-16 |
| π | OI | 8.12×10^-4^ | 5.16×10^-5^ | 2.20E-16 |
|  | OT | 5.25×10^-4^ | 7.58×10^-5^ | 2.20E-16 |
| Tajima's *D* | OI | 1.85 | 1.12 | 0.006899 |
|  | OT | 2.66 | 1.45 | 5.06E-08 |
| LD (*r^2^*) | OI | 0.18 | 0.46 | 0.107 |
|  | OT | 0.24 | 0.38 | 2.20E-16 |
| *ρ* | OI | 25.81 | 7.67 | 3.42E-05 |
|  | OT | 167.83 | 61.08 | 1.61E-04 |
| OK_OT | | | | |
| *F*_ST_ |  | 0.51 | 0.83 | 2.20E-16 |
| *D*xy |  | 0.47 | 0.67 | 2.20E-16 |
| π | OK | 9.48×10^-4^ | 9.01×10^-5^ | 2.20E-16 |
|  | OT | 5.25×10^-4^ | 7.58×10^-5^ | 2.20E-16 |
| Tajima's *D* | OK | 1.85 | 0.81 | 0.001034 |
|  | OT | 1.64 | 1.45 | 5.06E-08 |
| LD (*r^2^*) | OK | 0.13 | 0.39 | 5.26E-05 |
|  | OT | 0.24 | 0.38 | 2.20E-16 |
| *ρ* | OK | 32.92 | 6.00 | 3.82E-03 |
|  | OT | 167.83 | 24.98 | 5.54E-08 |

### Table S9. The number of PSGs between paired comparisons.

|  | **PSGs** | **PSG in islands** |
| --- | --- | --- |
| OI | 531 | 247 |
| OK | 534 | 186 |
| OT | 475 | 197 |
| Total | 1252 | 528 |

### Table S10. Results from Gene Ontology enrichment analysis for PSGs.

| **GO ID** | **GO Name** | **Ontology** | **PSGs** | **Annotated** | ***P*-value** |
| --- | --- | --- | --- | --- | --- |
| **OI** | | | | | |
| GO:0042802 | identical protein binding | Molecular function | 18 | 152 | 0.001378186 |
| GO:0005515 | protein binding | Molecular function | 43 | 152 | 0.003630003 |
| GO:0017111 | nucleoside-triphosphatase activity | Molecular function | 16 | 152 | 0.008760998 |
| GO:0016772 | transferase activity, transferring phosphorus-containing groups | Molecular function | 25 | 152 | 0.013212336 |
| GO:0016462 | pyrophosphatase activity | Molecular function | 16 | 152 | 0.015489722 |
| GO:0016818 | hydrolase activity, acting on acid anhydrides, in phosphorus-containing anhydrides | Molecular function | 16 | 152 | 0.016317669 |
| GO:0140101 | catalytic activity, acting on a tRNA | Molecular function | 5 | 152 | 0.016394806 |
| GO:0016817 | hydrolase activity, acting on acid anhydrides | Molecular function | 16 | 152 | 0.017179655 |
| GO:0016301 | kinase activity | Molecular function | 22 | 152 | 0.017525209 |
| GO:0140640 | catalytic activity, acting on a nucleic acid | Molecular function | 13 | 152 | 0.021954038 |
| GO:0004674 | protein serine/threonine kinase activity | Molecular function | 16 | 152 | 0.022568404 |
| GO:0051020 | GTPase binding | Molecular function | 5 | 152 | 0.026011991 |
| GO:0140657 | ATP-dependent activity | Molecular function | 13 | 152 | 0.034876346 |
| GO:0098772 | molecular function regulator | Molecular function | 9 | 152 | 0.036100693 |
| GO:0046983 | protein dimerization activity | Molecular function | 10 | 152 | 0.037426776 |
| GO:0016887 | ATP hydrolysis activity | Molecular function | 12 | 152 | 0.037971904 |
| GO:0042803 | protein homodimerization activity | Molecular function | 9 | 152 | 0.039107604 |
| GO:0004672 | protein kinase activity | Molecular function | 17 | 152 | 0.042402434 |
| GO:0015075 | ion transmembrane transporter activity | Molecular function | 14 | 152 | 0.044152576 |
| GO:0044451 | obsolete nucleoplasm part | Cellular component | 18 | 202 | 7.22E-04 |
| GO:0044425 | obsolete membrane part | Cellular component | 35 | 202 | 0.008820769 |
| GO:0005654 | nucleoplasm | Cellular component | 21 | 202 | 0.00913022 |
| GO:0044428 | obsolete nuclear part | Cellular component | 38 | 202 | 0.013948425 |
| GO:0044459 | obsolete plasma membrane part | Cellular component | 15 | 202 | 0.014123605 |
| GO:0005667 | transcription regulator complex | Cellular component | 7 | 202 | 0.014936139 |
| GO:0016604 | nuclear body | Cellular component | 7 | 202 | 0.020425547 |
| GO:0043233 | organelle lumen | Cellular component | 36 | 202 | 0.022301073 |
| GO:0031974 | membrane-enclosed lumen | Cellular component | 36 | 202 | 0.022301073 |
| GO:0070013 | intracellular organelle lumen | Cellular component | 36 | 202 | 0.022301073 |
| GO:0016591 | RNA polymerase II, holoenzyme | Cellular component | 5 | 202 | 0.026125562 |
| GO:0031981 | nuclear lumen | Cellular component | 32 | 202 | 0.029942211 |
| GO:0140513 | nuclear protein-containing complex | Cellular component | 20 | 202 | 0.037719515 |
| GO:0061695 | transferase complex, transferring phosphorus-containing groups | Cellular component | 7 | 202 | 0.047856263 |
| GO:0046496 | nicotinamide nucleotide metabolic process | Biological process | 6 | 193 | 0.001830099 |
| GO:0019362 | pyridine nucleotide metabolic process | Biological process | 6 | 193 | 0.002052618 |
| GO:0072524 | pyridine-containing compound metabolic process | Biological process | 6 | 193 | 0.002423993 |
| GO:0006733 | obsolete oxidoreduction coenzyme metabolic process | Biological process | 6 | 193 | 0.003152446 |
| GO:0009117 | nucleotide metabolic process | Biological process | 11 | 193 | 0.006298468 |
| GO:0006753 | nucleoside phosphate metabolic process | Biological process | 11 | 193 | 0.007066181 |
| GO:0051247 | positive regulation of protein metabolic process | Biological process | 9 | 193 | 0.011159588 |
| GO:0006366 | transcription by RNA polymerase II | Biological process | 15 | 193 | 0.012587968 |
| GO:0071482 | cellular response to light stimulus | Biological process | 6 | 193 | 0.013066939 |
| GO:0072594 | establishment of protein localization to organelle | Biological process | 10 | 193 | 0.013588549 |
| GO:0033365 | protein localization to organelle | Biological process | 11 | 193 | 0.020103109 |
| GO:0071478 | cellular response to radiation | Biological process | 6 | 193 | 0.021748867 |
| GO:0045944 | positive regulation of transcription by RNA polymerase II | Biological process | 7 | 193 | 0.021877094 |
| GO:0017038 | protein import | Biological process | 6 | 193 | 0.023054266 |
| GO:0006357 | regulation of transcription by RNA polymerase II | Biological process | 13 | 193 | 0.024448959 |
| GO:0009657 | plastid organization | Biological process | 9 | 193 | 0.026097808 |
| GO:0034968 | histone lysine methylation | Biological process | 5 | 193 | 0.028757208 |
| GO:0016571 | histone methylation | Biological process | 6 | 193 | 0.028800962 |
| GO:0055086 | nucleobase-containing small molecule metabolic process | Biological process | 11 | 193 | 0.030099646 |
| GO:0006281 | DNA repair | Biological process | 9 | 193 | 0.032697537 |
| GO:0031401 | positive regulation of protein modification process | Biological process | 6 | 193 | 0.033681922 |
| GO:0006886 | intracellular protein transport | Biological process | 13 | 193 | 0.034376369 |
| GO:0018022 | peptidyl-lysine methylation | Biological process | 5 | 193 | 0.035654753 |
| GO:0009314 | response to radiation | Biological process | 21 | 193 | 0.036107497 |
| GO:0006310 | DNA recombination | Biological process | 6 | 193 | 0.036311815 |
| GO:1901293 | nucleoside phosphate biosynthetic process | Biological process | 7 | 193 | 0.03821645 |
| GO:0009165 | nucleotide biosynthetic process | Biological process | 7 | 193 | 0.03821645 |
| GO:0008213 | protein alkylation | Biological process | 6 | 193 | 0.039070352 |
| GO:0006479 | protein methylation | Biological process | 6 | 193 | 0.039070352 |
| GO:0009658 | chloroplast organization | Biological process | 7 | 193 | 0.039862434 |
| GO:0010118 | stomatal movement | Biological process | 6 | 193 | 0.040981615 |
| GO:0048469 | cell maturation | Biological process | 6 | 193 | 0.041959076 |
| GO:0045859 | regulation of protein kinase activity | Biological process | 5 | 193 | 0.043493873 |
| GO:0006810 | transport | Biological process | 44 | 193 | 0.044483611 |
| GO:0019637 | organophosphate metabolic process | Biological process | 14 | 193 | 0.045467785 |
| GO:0016310 | phosphorylation | Biological process | 25 | 193 | 0.045615662 |
| **OK** | | | | | |
| GO:0022836 | gated channel activity | Molecular function | 6 | 171 | 3.68E-04 |
| GO:0022839 | ion gated channel activity | Molecular function | 5 | 171 | 0.001306807 |
| GO:0005216 | ion channel activity | Molecular function | 6 | 171 | 0.001595359 |
| GO:0000166 | nucleotide binding | Molecular function | 13 | 171 | 0.002322876 |
| GO:1901265 | nucleoside phosphate binding | Molecular function | 13 | 171 | 0.002322876 |
| GO:0042626 | ATPase-coupled transmembrane transporter activity | Molecular function | 8 | 171 | 0.00568825 |
| GO:0060589 | nucleoside-triphosphatase regulator activity | Molecular function | 6 | 171 | 0.008342904 |
| GO:0015399 | primary active transmembrane transporter activity | Molecular function | 8 | 171 | 0.009653442 |
| GO:0036094 | small molecule binding | Molecular function | 14 | 171 | 0.012089254 |
| GO:0022803 | passive transmembrane transporter activity | Molecular function | 6 | 171 | 0.014618246 |
| GO:0015267 | channel activity | Molecular function | 6 | 171 | 0.014618246 |
| GO:0050662 | obsolete coenzyme binding | Molecular function | 6 | 171 | 0.016731348 |
| GO:0022853 | active ion transmembrane transporter activity | Molecular function | 7 | 171 | 0.020778813 |
| GO:0030695 | GTPase regulator activity | Molecular function | 5 | 171 | 0.02158857 |
| GO:0008324 | cation transmembrane transporter activity | Molecular function | 11 | 171 | 0.021860077 |
| GO:0005524 | ATP binding | Molecular function | 6 | 171 | 0.026519155 |
| GO:0015075 | ion transmembrane transporter activity | Molecular function | 16 | 171 | 0.029100349 |
| GO:0032553 | ribonucleotide binding | Molecular function | 8 | 171 | 0.031619637 |
| GO:0022890 | inorganic cation transmembrane transporter activity | Molecular function | 10 | 171 | 0.033537822 |
| GO:0043168 | anion binding | Molecular function | 15 | 171 | 0.03378171 |
| GO:0046983 | protein dimerization activity | Molecular function | 11 | 171 | 0.034489539 |
| GO:0035639 | purine ribonucleoside triphosphate binding | Molecular function | 7 | 171 | 0.038942067 |
| GO:0140657 | ATP-dependent activity | Molecular function | 14 | 171 | 0.0402407 |
| GO:0022857 | transmembrane transporter activity | Molecular function | 22 | 171 | 0.044629043 |
| GO:0032559 | adenyl ribonucleotide binding | Molecular function | 6 | 171 | 0.048988383 |
| GO:0030554 | adenyl nucleotide binding | Molecular function | 6 | 171 | 0.048988383 |
| GO:0061695 | transferase complex, transferring phosphorus-containing groups | Cellular component | 9 | 215 | 0.008795618 |
| GO:0016020 | membrane | Cellular component | 93 | 215 | 0.021803339 |
| GO:0044425 | obsolete membrane part | Cellular component | 35 | 215 | 0.021857577 |
| GO:0016591 | RNA polymerase II, holoenzyme | Cellular component | 5 | 215 | 0.032942279 |
| GO:0055029 | nuclear DNA-directed RNA polymerase complex | Cellular component | 6 | 215 | 0.035365312 |
| GO:0031968 | organelle outer membrane | Cellular component | 5 | 215 | 0.036399293 |
| GO:0019867 | outer membrane | Cellular component | 5 | 215 | 0.036399293 |
| GO:0030880 | RNA polymerase complex | Cellular component | 6 | 215 | 0.048471422 |
| GO:0000428 | DNA-directed RNA polymerase complex | Cellular component | 6 | 215 | 0.048471422 |
| GO:0051130 | positive regulation of cellular component organization | Biological process | 11 | 210 | 4.13E-04 |
| GO:0009657 | plastid organization | Biological process | 12 | 210 | 0.002264989 |
| GO:0080135 | regulation of cellular response to stress | Biological process | 7 | 210 | 0.003077201 |
| GO:0044282 | small molecule catabolic process | Biological process | 8 | 210 | 0.009057411 |
| GO:0048511 | rhythmic process | Biological process | 7 | 210 | 0.010946838 |
| GO:0007623 | circadian rhythm | Biological process | 7 | 210 | 0.010946838 |
| GO:0045944 | positive regulation of transcription by RNA polymerase II | Biological process | 8 | 210 | 0.011202784 |
| GO:0046496 | nicotinamide nucleotide metabolic process | Biological process | 5 | 210 | 0.013201025 |
| GO:0010638 | positive regulation of organelle organization | Biological process | 6 | 210 | 0.01338218 |
| GO:0019362 | pyridine nucleotide metabolic process | Biological process | 5 | 210 | 0.014439273 |
| GO:0072524 | pyridine-containing compound metabolic process | Biological process | 5 | 210 | 0.016439169 |
| GO:0006733 | obsolete oxidoreduction coenzyme metabolic process | Biological process | 5 | 210 | 0.020166996 |
| GO:0046395 | carboxylic acid catabolic process | Biological process | 6 | 210 | 0.021710925 |
| GO:0016054 | organic acid catabolic process | Biological process | 6 | 210 | 0.021710925 |
| GO:0019637 | organophosphate metabolic process | Biological process | 16 | 210 | 0.022989913 |
| GO:0046907 | intracellular transport | Biological process | 20 | 210 | 0.024487126 |
| GO:0006796 | phosphate-containing compound metabolic process | Biological process | 39 | 210 | 0.030220415 |
| GO:0017038 | protein import | Biological process | 6 | 210 | 0.03298998 |
| GO:0006793 | phosphorus metabolic process | Biological process | 39 | 210 | 0.038050538 |
| GO:0051649 | establishment of localization in cell | Biological process | 21 | 210 | 0.038605541 |
| GO:0034968 | histone lysine methylation | Biological process | 5 | 210 | 0.039194895 |
| GO:0006357 | regulation of transcription by RNA polymerase II | Biological process | 13 | 210 | 0.043854468 |
| GO:0006650 | glycerophospholipid metabolic process | Biological process | 5 | 210 | 0.045555839 |
| GO:0051247 | positive regulation of protein metabolic process | Biological process | 8 | 210 | 0.046200575 |
| GO:0031401 | positive regulation of protein modification process | Biological process | 6 | 210 | 0.047521953 |
| GO:0098655 | cation transmembrane transport | Biological process | 12 | 210 | 0.048054209 |
| GO:0018022 | peptidyl-lysine methylation | Biological process | 5 | 210 | 0.04826293 |
| GO:0006366 | transcription by RNA polymerase II | Biological process | 14 | 210 | 0.048611726 |
| **OT** | | | | | |
| GO:0140101 | catalytic activity, acting on a tRNA | Molecular function | 8 | 181 | 3.81E-04 |
| GO:0019901 | protein kinase binding | Molecular function | 7 | 181 | 0.001558373 |
| GO:0019900 | kinase binding | Molecular function | 7 | 181 | 0.003155954 |
| GO:0042626 | ATPase-coupled transmembrane transporter activity | Molecular function | 8 | 181 | 0.00792877 |
| GO:0015399 | primary active transmembrane transporter activity | Molecular function | 8 | 181 | 0.013286949 |
| GO:0016874 | ligase activity | Molecular function | 7 | 181 | 0.027293303 |
| GO:0004519 | endonuclease activity | Molecular function | 14 | 181 | 0.036935881 |
| GO:0140640 | catalytic activity, acting on a nucleic acid | Molecular function | 14 | 181 | 0.03796795 |
| GO:0140098 | catalytic activity, acting on RNA | Molecular function | 11 | 181 | 0.04868297 |
| GO:0046527 | glucosyltransferase activity | Molecular function | 6 | 181 | 0.04971761 |
| GO:0005654 | nucleoplasm | Cellular component | 24 | 213 | 0.001972845 |
| GO:0140513 | nuclear protein-containing complex | Cellular component | 25 | 213 | 0.002761577 |
| GO:0044451 | obsolete nucleoplasm part | Cellular component | 17 | 213 | 0.003262863 |
| GO:0032991 | protein-containing complex | Cellular component | 59 | 213 | 0.003411871 |
| GO:0090575 | RNA polymerase II transcription regulator complex | Cellular component | 5 | 213 | 0.005776015 |
| GO:0005667 | transcription regulator complex | Cellular component | 8 | 213 | 0.005984936 |
| GO:1990234 | transferase complex | Cellular component | 17 | 213 | 0.006831858 |
| GO:0044454 | obsolete nuclear chromosome part | Cellular component | 10 | 213 | 0.008145059 |
| GO:0140535 | intracellular protein-containing complex | Cellular component | 18 | 213 | 0.009343959 |
| GO:0000228 | nuclear chromosome | Cellular component | 10 | 213 | 0.01026834 |
| GO:0044427 | obsolete chromosomal part | Cellular component | 14 | 213 | 0.012735136 |
| GO:0016607 | nuclear speck | Cellular component | 6 | 213 | 0.012735668 |
| GO:0016021 | integral component of membrane | Cellular component | 20 | 213 | 0.014478152 |
| GO:0044424 | obsolete intracellular part | Cellular component | 195 | 213 | 0.014569392 |
| GO:1902494 | catalytic complex | Cellular component | 25 | 213 | 0.018076461 |
| GO:0044428 | obsolete nuclear part | Cellular component | 39 | 213 | 0.019540539 |
| GO:0031981 | nuclear lumen | Cellular component | 34 | 213 | 0.023393103 |
| GO:0061695 | transferase complex, transferring phosphorus-containing groups | Cellular component | 8 | 213 | 0.023665812 |
| GO:0005622 | intracellular anatomical structure | Cellular component | 195 | 213 | 0.024609447 |
| GO:0016604 | nuclear body | Cellular component | 7 | 213 | 0.026343593 |
| GO:0005694 | chromosome | Cellular component | 14 | 213 | 0.033508021 |
| GO:0055029 | nuclear DNA-directed RNA polymerase complex | Cellular component | 6 | 213 | 0.034021726 |
| GO:1990904 | ribonucleoprotein complex | Cellular component | 17 | 213 | 0.046130484 |
| GO:0030880 | RNA polymerase complex | Cellular component | 6 | 213 | 0.04669588 |
| GO:0000428 | DNA-directed RNA polymerase complex | Cellular component | 6 | 213 | 0.04669588 |
| GO:0000785 | chromatin | Cellular component | 8 | 213 | 0.047572142 |
| GO:0007417 | central nervous system development | Biological process | 5 | 211 | 3.28E-04 |
| GO:0044728 | DNA methylation or demethylation | Biological process | 7 | 211 | 3.90E-04 |
| GO:0006139 | nucleobase-containing compound metabolic process | Biological process | 94 | 211 | 4.98E-04 |
| GO:1901360 | organic cyclic compound metabolic process | Biological process | 103 | 211 | 6.03E-04 |
| GO:0006304 | DNA modification | Biological process | 7 | 211 | 7.22E-04 |
| GO:0030182 | neuron differentiation | Biological process | 6 | 211 | 9.46E-04 |
| GO:0006305 | DNA alkylation | Biological process | 6 | 211 | 0.00109498 |
| GO:0006306 | DNA methylation | Biological process | 6 | 211 | 0.00109498 |
| GO:0043412 | macromolecule modification | Biological process | 64 | 211 | 0.001202879 |
| GO:0006399 | tRNA metabolic process | Biological process | 9 | 211 | 0.001299793 |
| GO:0048699 | generation of neurons | Biological process | 6 | 211 | 0.001351605 |
| GO:0006725 | cellular aromatic compound metabolic process | Biological process | 99 | 211 | 0.001445851 |
| GO:0046483 | heterocycle metabolic process | Biological process | 97 | 211 | 0.001554045 |
| GO:0044786 | cell cycle DNA replication | Biological process | 6 | 211 | 0.001878386 |
| GO:0043414 | macromolecule methylation | Biological process | 12 | 211 | 0.001978976 |
| GO:0022008 | neurogenesis | Biological process | 6 | 211 | 0.002000142 |
| GO:0034641 | cellular nitrogen compound metabolic process | Biological process | 101 | 211 | 0.002026187 |
| GO:0007399 | nervous system development | Biological process | 7 | 211 | 0.002487665 |
| GO:0032259 | methylation | Biological process | 14 | 211 | 0.002826609 |
| GO:0009451 | RNA modification | Biological process | 19 | 211 | 0.002935589 |
| GO:0090304 | nucleic acid metabolic process | Biological process | 84 | 211 | 0.002989736 |
| GO:0006323 | DNA packaging | Biological process | 9 | 211 | 0.003295545 |
| GO:0006400 | tRNA modification | Biological process | 5 | 211 | 0.005230191 |
| GO:0016569 | obsolete covalent chromatin modification | Biological process | 10 | 211 | 0.005815821 |
| GO:0042592 | homeostatic process | Biological process | 17 | 211 | 0.005909512 |
| GO:0006259 | DNA metabolic process | Biological process | 19 | 211 | 0.006035111 |
| GO:0071103 | DNA conformation change | Biological process | 10 | 211 | 0.006325101 |
| GO:0006325 | chromatin organization | Biological process | 18 | 211 | 0.006879708 |
| GO:0006396 | RNA processing | Biological process | 22 | 211 | 0.007492018 |
| GO:0016070 | RNA metabolic process | Biological process | 75 | 211 | 0.007554806 |
| GO:0016458 | obsolete gene silencing | Biological process | 9 | 211 | 0.007828585 |
| GO:0051276 | chromosome organization | Biological process | 23 | 211 | 0.008125037 |
| GO:0060968 | obsolete regulation of gene silencing | Biological process | 5 | 211 | 0.00961877 |
| GO:0000398 | mRNA splicing, via spliceosome | Biological process | 8 | 211 | 0.010520004 |
| GO:0006807 | nitrogen compound metabolic process | Biological process | 136 | 211 | 0.010581495 |
| GO:0080135 | regulation of cellular response to stress | Biological process | 6 | 211 | 0.012681396 |
| GO:0046496 | nicotinamide nucleotide metabolic process | Biological process | 5 | 211 | 0.013452011 |
| GO:0044238 | primary metabolic process | Biological process | 147 | 211 | 0.013490003 |
| GO:0040029 | regulation of gene expression, epigenetic | Biological process | 10 | 211 | 0.014509896 |
| GO:0019362 | pyridine nucleotide metabolic process | Biological process | 5 | 211 | 0.014712048 |
| GO:0006397 | mRNA processing | Biological process | 10 | 211 | 0.014843714 |
| GO:0008380 | RNA splicing | Biological process | 10 | 211 | 0.015183242 |
| GO:0072524 | pyridine-containing compound metabolic process | Biological process | 5 | 211 | 0.016746739 |
| GO:0009657 | plastid organization | Biological process | 10 | 211 | 0.017724567 |
| GO:0008033 | tRNA processing | Biological process | 5 | 211 | 0.018202236 |
| GO:0016071 | mRNA metabolic process | Biological process | 14 | 211 | 0.018530319 |
| GO:0016570 | histone modification | Biological process | 10 | 211 | 0.018904968 |
| GO:0071482 | cellular response to light stimulus | Biological process | 6 | 211 | 0.019447617 |
| GO:1901419 | regulation of response to alcohol | Biological process | 7 | 211 | 0.019645467 |
| GO:1905957 | regulation of cellular response to alcohol | Biological process | 7 | 211 | 0.019645467 |
| GO:0009787 | regulation of abscisic acid-activated signaling pathway | Biological process | 7 | 211 | 0.019645467 |
| GO:0070828 | heterochromatin organization | Biological process | 6 | 211 | 0.020103968 |
| GO:0031507 | heterochromatin assembly | Biological process | 6 | 211 | 0.020103968 |
| GO:0006733 | obsolete oxidoreduction coenzyme metabolic process | Biological process | 5 | 211 | 0.020538235 |
| GO:0048583 | regulation of response to stimulus | Biological process | 28 | 211 | 0.02067176 |
| GO:0018393 | internal peptidyl-lysine acetylation | Biological process | 5 | 211 | 0.021358423 |
| GO:0018394 | peptidyl-lysine acetylation | Biological process | 5 | 211 | 0.021358423 |
| GO:0006475 | internal protein amino acid acetylation | Biological process | 5 | 211 | 0.021358423 |
| GO:0034660 | ncRNA metabolic process | Biological process | 13 | 211 | 0.022988791 |
| GO:0009658 | chloroplast organization | Biological process | 8 | 211 | 0.023087061 |
| GO:0010467 | gene expression | Biological process | 71 | 211 | 0.025048269 |
| GO:0000377 | RNA splicing, via transesterification reactions with bulged adenosine as nucleophile | Biological process | 8 | 211 | 0.025410556 |
| GO:0048878 | chemical homeostasis | Biological process | 13 | 211 | 0.026596607 |
| GO:0000375 | RNA splicing, via transesterification reactions | Biological process | 8 | 211 | 0.026631616 |
| GO:0006261 | DNA-dependent DNA replication | Biological process | 7 | 211 | 0.027981212 |
| GO:0031047 | gene silencing by RNA | Biological process | 6 | 211 | 0.030041803 |
| GO:0034285 | response to disaccharide | Biological process | 5 | 211 | 0.030746364 |
| GO:0009744 | response to sucrose | Biological process | 5 | 211 | 0.030746364 |
| GO:0006473 | protein acetylation | Biological process | 5 | 211 | 0.030746364 |
| GO:0071478 | cellular response to radiation | Biological process | 6 | 211 | 0.031811964 |
| GO:0045814 | negative regulation of gene expression, epigenetic | Biological process | 6 | 211 | 0.032722029 |
| GO:0043170 | macromolecule metabolic process | Biological process | 125 | 211 | 0.036541234 |
| GO:0006974 | cellular response to DNA damage stimulus | Biological process | 11 | 211 | 0.039156565 |
| GO:0051052 | regulation of DNA metabolic process | Biological process | 7 | 211 | 0.039343949 |
| GO:0071704 | organic substance metabolic process | Biological process | 152 | 211 | 0.041314577 |
| GO:0043543 | protein acylation | Biological process | 5 | 211 | 0.042384926 |
| GO:0009987 | cellular process | Biological process | 195 | 211 | 0.043412375 |
| GO:0065008 | regulation of biological quality | Biological process | 30 | 211 | 0.04433731 |
| GO:0030001 | metal ion transport | Biological process | 9 | 211 | 0.045617943 |
| GO:0019637 | organophosphate metabolic process | Biological process | 15 | 211 | 0.045632898 |
| GO:0010629 | negative regulation of gene expression | Biological process | 18 | 211 | 0.045972377 |
| GO:0006650 | glycerophospholipid metabolic process | Biological process | 5 | 211 | 0.046329707 |

### Table S11. List of some PSGs with putative functions associated with ecological adaptation and morphological divergence in the three species.

| **Gene ID** | **Uniprot ID** | **Gene Name** | **Selected in** |
| --- | --- | --- | --- |
| *Genes associated with drought tolerance* | | | |
| OthT040571.1 | Q9ZT50 | *RHA2A* | OT, OK, OI |
| OthT024551.1 | Q9M022 | *AIRP2* | OT, OK, OI |
| OthT024637.1 | Q8H1H9 | *CuAOgamma1* | OK, OI |
| OthT015453.1 | Q9C8E6 | *PMI1* | OK |
| OthT031192.1 | Q6L4D2 | *PM19L* | OK |
| OthT024601.1 | Q9C5Y0 | *PLDD1* | OI |
| *Genes involved in light response* | | | |
| OthT041013.1 | Q7X6P3 | *RUS1* | OT, OI |
| OthT012361.1 | Q9M7I9 | *STEP1* | OT, OI |
| OthT039226.1 | O81208 | *OHP1* | OT |
| OthT012088.1 | Q9C5J9 | *LIIP1* | OK |
| *Genes related to salt tolerance* | | | |
| OthT012088.1 | Q9C5J9 | *LIIP1* | OK |
| OthT002715.1 | Q336R9 | *MSRA4* | OI |
| OthT043427.1 | Q8RXY6 | *SGF29A* | OI |
| OthT029858.1 | Q948T6 | *LGUL* | OI |
| *Genes associated with flowering time* | | | |
| OthT040541.1 | E5RQA1 | *GHD7* | OT |
| OthT002289.1 | Q9SUF1 | *WTR31* | OK |
| *Genes associated with DNA damage repair* | | | |
| OthT047727.1 | F4JEX5 | *FIGL1* | OT |
| OthT018447.1 | Q9SJB3 | *PMA5* | OK |
| *Genes involved in pathogen stress* | | | |
| OthT022359.1 | F2VYU4 | *PIK1* | OT |
| OthT035856.1 | A0A3Q7ELQ2 | *MTB1* | OK |
| OthT026994.1 | A5H452 | *PER70* | OI |
| *Genes associated with leaf and shoot development* | | | |
| OthT008449.1 | Q9AQW1 | *REL2* | OT |
| OthT018347.1 | Q42485 | *ZFP1* | OT |
| OthT030831.1 | P38666 | *RL242* | OI |
| *Genes associated with pollen development* | | | |
| OthT031133.1 | Q8LDM2 | *PKSB* | OK |
| *Genes associated with fruit and seed development* | | | |
| OthT048103.1 | Q9FIN7 | *MIP2* | OT |
| OthT008485.1 | Q9ZNU6 | *DET1* | OI |
